# Effectiveness of Digital Lifestyle Interventions on Depression, Anxiety, Stress, and Well-Being: Systematic Review and Meta-Analysis

**DOI:** 10.2196/56975

**Published:** 2025-03-20

**Authors:** Jacinta Brinsley, Edward J O'Connor, Ben Singh, Grace McKeon, Rachel Curtis, Ty Ferguson, Georgia Gosse, Iris Willems, Pieter-Jan Marent, Kimberley Szeto, Joseph Firth, Carol Maher

**Affiliations:** 1 Alliance for Research in Exercise, Nutrition and Activity University of South Australia Adelaide Australia; 2 Health & Biosecurity Commonwealth Scientific and Industrial Research Organisation (CSIRO) Adelaide Australia; 3 Discipline of Psychiatry and Mental Health, School of Clinical Medicine Nutrition, Exercise and Social Equity (NExuS) University of New South Wales Sydney Australia; 4 IIMPACT in Health University of South Australia Adelaide Australia; 5 Department of Movement and Sports Sciences Research Centre for Aging Young Ghent University Ghent Belgium; 6 Research Foundation Flanders Brussels Belgium; 7 Department of Movement Sciences, KU Leuven Leuven Belgium; 8 Division of Psychology and Mental Health Manchester Academic Health Science Centre The University of Manchester Manchester United Kingdom; 9 Manchester Academic Health Science Centre Greater Manchester Mental Health NHS Foundation Trust Manchester United Kingdom

**Keywords:** depression, anxiety, stress, well-being, mental health, lifestyle intervention, physical activity, sleep, diet, digital health, mobile phone

## Abstract

**Background:**

There is a growing body of robust evidence to show that lifestyle behaviors influence mental health outcomes. Technology offers an accessible and cost-effective implementation method for interventions, yet the study of the effectiveness of interventions to date has been specific to the mode of delivery, population, or behavior.

**Objective:**

The primary aim of this review was to comprehensively evaluate the effectiveness of digital lifestyle interventions for improving symptoms of depression, anxiety, stress, and well-being as coprimary outcomes in adults. The secondary aim was to explore the technological, methodological, intervention-specific, and population-specific characteristics that were associated with major changes in mental health outcomes.

**Methods:**

A systematic search was conducted across the MEDLINE, CINAHL, Embase, Emcare, PsycINFO, and Scopus databases to identify studies published between January 2013 and January 2023. Randomized controlled trials of lifestyle interventions (physical activity, sleep, and diet) that were delivered digitally; reported changes in symptoms of depression, anxiety, stress, or well-being in adults (aged ≥18 years); and were published in English were included. Multiple authors independently extracted data, which was evaluated using the 2011 Levels of Evidence from the Oxford Centre for Evidence-Based Medicine. Inverse-variance random-effects meta-analyses were used for data analysis. The primary outcome was the change in symptoms of depression, anxiety, stress, and well-being as measured by validated self-report of clinician-administered outcomes from pre- to postintervention. Subgroup analyses were conducted to determine whether results differed based on the target lifestyle behavior, delivery method, digital features, design features, or population characteristics.

**Results:**

Of the 14,356 studies identified, 61 (0.42%) were included. Digital lifestyle interventions had a significant small-to-medium effect on depression (standardized mean difference [SMD] −0.37; *P*<.001), a small effect on anxiety (SMD −0.29; *P<*.001) and stress (SMD −0.17; *P*=.04), and no effect on well-being (SMD 0.14; *P*=.15). Subgroup analyses generally suggested that effects were similar regardless of the delivery method or features used, the duration and frequency of the intervention, the population, or the lifestyle behavior targeted.

**Conclusions:**

Overall, these results indicate that delivering lifestyle interventions via a range of digital methods can have significant positive effects on depression (*P*<.001), anxiety (*P*<.001), and stress (*P*=.04) for a broad range of populations, while effects on well-being are inconclusive. Future research should explore how these interventions can be effectively implemented and embedded within health care with a concerted focus on addressing digital health equity.

**Trial Registration:**

PROSPERO CRD42023428908; https://www.crd.york.ac.uk/PROSPERO/view/CRD42023428908

## Introduction

### Background

Mental disorders are among the leading causes of global disease burden [1] and a significant risk factor for premature mortality [2]. Recent estimates suggest that between 5% and 19% of global disability-adjusted life-years can be attributed to mental disorders [1,3], causing annual economic losses of approximately US $4.7 trillion [4]. These mental health difficulties cause enormous psychological impact, have widespread deleterious effects on health behaviors [5], and vastly increase the odds of experiencing physical health morbidities [6].

There is increasing recognition that poor lifestyle behaviors, such as diet, physical activity, and sleep, contribute to both poor physical and mental health. Concurrently, there is increasing evidence for the role of lifestyle interventions in preventing, managing, and treating mental illness [7-9]. Numerous clinical guidelines and health policy documents now call for lifestyle behaviors to be addressed alongside pharmacotherapies and psychotherapy, as part of mental health care [10].

Digital technology offers a promising avenue for revolutionizing mental health care delivery on a global scale. Digital interventions can also reduce the stigma associated with seeking help [11], while data-driven insights allow for personalized and efficient interventions tailored to individual needs.

In recent years, an increasing number of studies have demonstrated the effectiveness of digital interventions in delivering mental health treatments [12], including compelling outcomes for smartphone-delivered psychological interventions in reducing depression and anxiety symptoms [13,14]. Concurrently, research related to digital lifestyle interventions is increasing rapidly [15]. A growing body of evidence shows that these interventions can be effective for improving health behaviors such as physical activity [16], sleep quality [17], weight loss [18], and healthy diet [19]. However, there is little evidence specifically focused on the effect of digital lifestyle interventions on mental health and well-being outcomes in the general population. The few reviews in this area have focused on singular lifestyle behaviors such as physical activity [20], specific clinical populations [21], or a single delivery method [22] or have reported lifestyle behavior changes but not changes in mental health [19,23]. Moreover, only 1 review has synthesized data from >10 randomized controlled trials (RCTs) [19]. This fragmented approach limits our ability to rigorously discern the effectiveness of the various intervention components and combinations on mental health across the general population.

### Objectives

This systematic review and meta-analysis takes a broad approach, aiming to provide a thorough synthesis of the evidence on digitally delivered lifestyle interventions for depression, anxiety, stress, and well-being outcomes in adults. Recognizing that mental health is more than the absence of disorders and distress [24], we also evaluated the effectiveness of digital lifestyle interventions on psychological well-being (ie, well-being), as operationalized by van Agteren et al [25]. Here, well-being refers to positive aspects of mental health, including positive affect and life satisfaction (ie, subjective well-being), as well as meaning, purpose, and related concepts (ie, psychological well-being).

Given the breadth of the review, the secondary aim was to explore the technology-specific intervention features that are associated with greater mental health outcomes. We further considered the impact of various methodological (ie, study quality), intervention-specific (ie, type, delivery method, and delivery features), and population-specific (ie, general population, those with a mental illness, and those with a physical illness) characteristics. Finally, this review aimed to shed light on the overall quality of the evidence provided in the meta-analyses and discuss the implications of the evidence for future research and lifestyle intervention delivery.

## Methods

This systematic review and meta-analysis adheres to the PRISMA (Preferred Reporting Items for Systematic Reviews and Meta-Analyses) guidelines [26] and is registered with PROSPERO (CRD42023428908). A completed PRISMA checklist for this study is available in Multimedia Appendix 1.

### Search Strategy and Selection of Studies

The following electronic databases were searched from January 2013 to January 10, 2023: MEDLINE (Ovid), CINAHL (EBSCOhost), Embase (Ovid), Emcare (Ovid), PsycINFO (Ovid), and Scopus. Subject heading, keyword, and Medical Subject Headings term searches relating to technology, modifiable lifestyle behaviors (ie, physical activity, diet, and sleep), mental health, and study design were used. Full search queries are detailed in Multimedia Appendix 2. Additional searches were conducted using Google Scholar and by scanning the reference lists of included papers and review articles to identify any additional studies. Studies were exported to Covidence (Veritas Health Innovation Ltd), and duplicates were removed. Studies were screened in duplicate with conflicts resolved between the 2 reviewers or a third independent reviewer.

### Inclusion and Exclusion Criteria

To be included in this systematic review and meta-analysis, studies needed to meet the following criteria: (1) involve adults (aged ≥18 years) of any health status and (2) include a digitally delivered lifestyle intervention targeting physical activity, diet, sleep, or any combination thereof. We defined digital lifestyle interventions as interventions delivered via digital technologies (such as apps, websites, and wearable devices) that were self-guided and did not require real-time clinician delivery (eg, behavior change and education) and aimed to improve lifestyle behaviors [27]. Considering the nature of the research, we included interventions that were supplemented with contact for follow-up or adherence and technology support (eg, orientation session or phone call to create a user profile or a peer-driven social forum moderated by a clinician). Lifestyle interventions were eligible if at least 50% of the intervention was focused on physical activity, diet, and sleep. Physical activity was considered in the broadest sense, including overall physical activity levels, structured exercise interventions, and studies examining reducing physical inactivity (ie, sedentary time). Diet was considered as any intervention targeting food intake (ie, amount or type). Studies focusing on supplementation or specific nutrient treatments were excluded. Sleep interventions were considered as anything targeting sleep; therefore, psychological therapies targeting sleep (eg, cognitive behavioral therapy for insomnia) were eligible for inclusion; (3) studies that had an adequate control condition of no treatment, treatment as usual, waitlist control, or attention (ie, sham) control were included, while control groups that received an alternative intervention comprising behavior change techniques or an in-person version of the digital intervention were excluded, as they would limit our ability to evaluate the effectiveness of the intervention; (4) studies that reported changes in symptoms of depression, anxiety, psychological stress, or well-being using validated tools were included, and due to the broad and varied definitions of well-being [28], we considered well-being measures listed as per the review by van Agteren et al [25]. Studies that focused on momentary affective states were considered outside the scope of this review and were excluded; (5) RCTs were included. No restriction was placed on the setting or context of the included studies.

### Data Synthesis and Analysis

In total, 2 reviewers independently extracted data using a predefined data extraction sheet, cross-checked the data, assessed study quality, and resolved disagreements by discussion or referral to a third reviewer (JB or CM). The authors of the included studies were contacted to provide additional data for inclusion in the meta-analysis if required.

Intervention effect sizes (ie, differences between intervention and control groups) for outcome data were calculated across all studies and standardized to Hedges *g* [29] along with the SE. Studies were grouped by outcomes (ie, depression, anxiety, stress, and well-being) for analyses. Random-effects meta-analyses were conducted due to expected heterogeneity. All analyses used an inverse variance method with restricted maximum-likelihood estimator for τ^2^ and Hartung-Knapp adjustment for the random effects model. Standardized mean differences (SMDs) were used as the effect measure for meta-analyses to allow comparison of data from different scales. If means and SDs were not reported in a study, authors were contacted twice before means and SDs were calculated based on available data using recommended formulas (eg, using sample size, median, and range) [30]. Meta-analyses were conducted only when at least 5 studies were included. Effect sizes were categorized as small (0.2), medium (0.5), or large (≥0.8) [31], with a significance level set at .05 [32]. Cochrane *Q* test was used to assess statistical heterogeneity, and the *I*^2^ statistic was used to quantify the proportion of the overall outcome effect attributed to heterogeneity. The following cutoff values for the *I*^2^ statistic were used: 0%-29%=no heterogeneity, 30%-49%=moderate heterogeneity, 50%-74%=substantial heterogeneity, and 75%-100%=considerable heterogeneity [33]. Publication bias was assessed with visual inspection of funnel plots and tested using the Begg-Mazumdar [34] and Egger regression method [35], with a *P* value <.05 suggesting the presence of bias. Where significant bias was detected, a Duval and Tweedie [36] trim-and-fill analysis was applied. Heterogeneity was explored with subgroup and sensitivity analyses. Sensitivity analyses were conducted by removing outliers, studies with samples <100, studies of poor to fair quality, and those with attrition >25% to investigate changes in effect for each outcome. All analyses were conducted in R software (version 4.2.0, “Mountain Hydrangea”; R Foundation for Statistical Computing).

### Quality Assessment

The risk of bias of each study was assessed using the Physiotherapy Evidence Database Scale (PEDro) [37]. The PEDro scale comprises 10 questions, each answered as yes or no. A study was deemed to be high quality with a score of 6 to 10, fair quality with a score of 4 to 5, and low quality with a score <4.

The overall level of evidence was graded using the Oxford Centre for Evidence-Based Medicine 2011 Levels of Evidence [38] as follows: grade A—consistent level 1 studies (ie, individual RCTs with narrow CIs), grade B—consistent level 2 (ie, individual cohort studies or low-quality RCTs) or level 3 studies (ie, individual case-control studies) or extrapolations from level 1 studies, grade C—level 4 studies or extrapolations from level 2 or 3 studies, or grade D—level 5 (ie, expert opinion without explicit critical appraisal) evidence or inconsistent or inconclusive studies of any level.

### Subgroup Analyses

The central aim of this review was to examine the effect of various methodological, intervention-specific, and population-specific factors on the effectiveness of digital lifestyle interventions for mental health outcomes [39]. Intervention-specific factors included the lifestyle behavior; the intervention targeted (ie, physical activity alone, diet alone, sleep alone, herein referred to as the respective singular behavior, or any combination of these behaviors, which is referred to herein as multibehavioral); delivery method (ie, web based, app, email or text, and wearable device); features of delivery format (ie, use of a chatbot or gamification); frequency and duration of the intervention; whether the intervention was co-designed, individualized, or publicly available; and whether it contained a theoretical underpinning. The frequency of intervention delivery was split into three categories: (1) intended daily use; (2) 1 to 4 times per week; and (3) “other,” which included fortnightly, monthly, varied, once-off, and self-paced engagement. Methodological factors were control group types and study quality. Study quality was based on the risk of bias, where studies were categorized into poor, fair, good, or excellent as per the PEDro scoring. Control groups were divided into no treatment, including waitlist; treatment as usual; or attention control. To determine which populations might benefit most from digital lifestyle interventions, we conducted subgroup analyses for each outcome for apparently healthy adults; those with a physical health condition; those diagnosed with a mental health condition; and, due to the number of sleep-specific studies, people who met the criteria for insomnia.

### Deviation From the Registered Protocol

We originally planned to include interventions targeting alcohol, smoking, and substance use as behaviors, considering their association with poor mental health outcomes. However, due to the unfeasible volume of search results and their distinct focus on addiction, these lifestyle behaviors were excluded from this review.

## Results

### Included Studies

**Figure 1:**
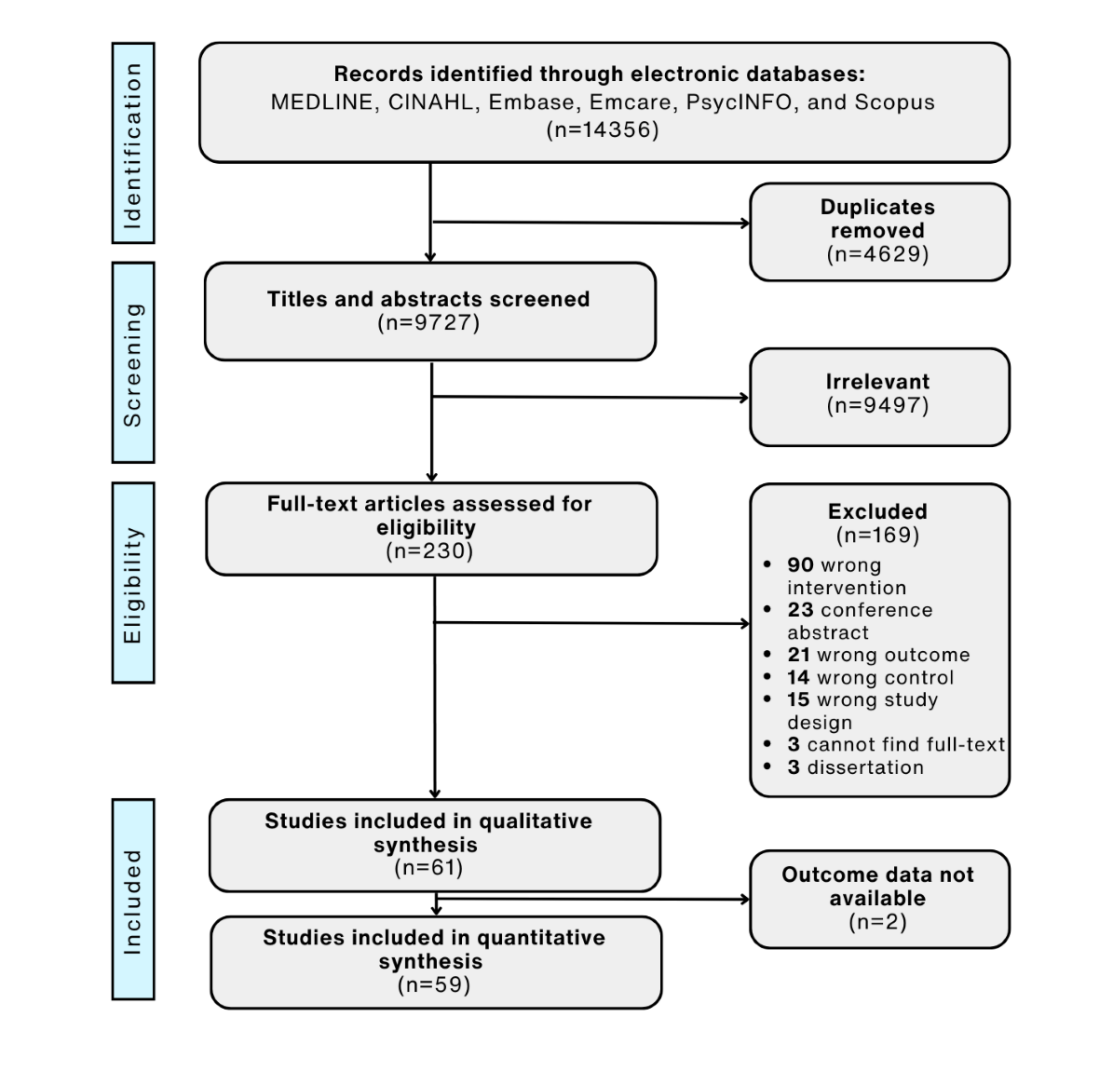
PRISMA flowchart of study selection.

Searching of databases yielded 14,357 results. Following the removal of duplicates, 9727 potentially eligible studies remained for which abstracts were screened. At full-text stage, 230 studies were reviewed, and 169 were removed because they failed to meet the inclusion criteria (Multimedia Appendix 3). The remaining 61 studies were included in the review, and 59 studies were included in the quantitative synthesis (Figure 1).

### Study Characteristics

In total, 22,483 participants across 61 studies were included in the review. Sample sizes ranged between 20 and 3755 participants, and the mean age ranged between 19 and 68 years. Interventions lasted between 2 and 52 weeks. Studies originated from Europe, America, Canada, Australia, New Zealand, the United Kingdom, Iran, and Asia. Full details of each study are outlined in Table 1.

**Table 1:** Descriptive characteristics of the included studies.

Study	Country	Sample population; N; female (%)	Intervention							Mental health outcome		Publicly available		Risk of bias, PEDro^a^ score
			Study aim	Behaviors targeted	Delivery format	Comparator	Duration (wk)	Included BCTs^b^						
Abbott et al [40], 2021	United States	Individuals experiencing major depressive disorder; 71; 86%	Efficacy for depressive symptoms	PA^c^, diet, and sleep	Web based	Waitlist	6	No	PHQ-9^d^ (secondary)		No		6	
Abedi et al [41], 2015	Iran	Postmenopausal women; 106; 100%	Effectiveness for depression, anxiety, and insomnia	PA	Wearable	No treatment	12	Yes	BDI^e^ (primary)		No		6	
Bade et al [42], 2021	United States	Individuals with advanced-stage lung cancer; 40; 75%	Feasibility, effectiveness for PA, QoL^f^, depression	PA	Wearable, app, and SMS text messages	Treatment as usual	12	Yes	PHQ-9 (secondary)		No		7	
Bailey et al [43], 2020	United Kingdom	Individuals with diabetes; 20; 50%	Feasibility	PA and sleep	App	Treatment as usual	8	Yes	WEMWBS^g^ (secondary)		No		7	
Bennion et al [44], 2020	United States	Low-income postpartum women; 370; 100%	Effectiveness for weight loss	PA and diet	Web based and SMS text messages	Treatment as usual	52	Yes	EPDS^h^ (secondary)		No		7	
Bossen et al [45], 2013	Netherlands	Individuals with hip and knee OA^i^; 199; 65%	Effectiveness for PA	PA	Web based and emails	Waitlist	9	Yes	HADS^j^ (secondary)		Yes; Join2Move		7	
Brindal et al [46], 2019	Australia	Individuals who had 5% weight loss in past 2 years; 88; 75%	Effectiveness for weight, food, exercise, mood, and stress	PA and diet	App	Attention control	12	Yes	DASS^k^-21 and SLS^l^-5 (primary)		Yes: Moti-Mate		7	
Carli et al [47], 2022	Italy and Portugal	Individuals with somatic disorders; 425; 44%	Effectiveness for depressive symptoms	PA, diet, and sleep	App and sensorized shirt	Treatment as usual	12	Yes	BDI-II (primary)		Yes; NEVERMIND		8	
Chee et al [48], 2016	United States	Asian American women with depressive symptoms; 33; 100%	Preliminary efficacy for depressive symptoms	PA	Web based	No treatment	12	Yes	CES-D^m^ (primary)		No		5	
Cheng et al [49], 2019	United States	Individuals experiencing insomnia; 1358; 38%	Efficacy for depression	Sleep	Web based and emails	Attention control	12	Yes	CES-D (primary)		Yes; Sleepio		9	
Christensen et al [50], 2016	Australia	Individuals experiencing insomnia and depressive symptoms; 1149; 74%	Effectiveness for depressive symptoms	Sleep	App and emails	Attention control	6	Yes	PHQ-9 (primary)		Yes; Sleepio		9	
Conner et al [51], 2017	New Zealand	Young adults; 115; 67%	Effectiveness for fruit or vegetable consumption	Diet	SMS text messages	No treatment	2	No	CES-D and HADS (primary)		No		7	
Devi et al [52], 2014	United Kingdom	Individuals with stable angina; 95; 26%	Effectiveness for cardiac rehabilitation	PA and diet	Website	Treatment as usual	6	Yes	HADS (secondary)		Yes; ActivateYourHeart		8	
Duan et al [53], 2017	China	University students; 493; 71%	Effectiveness for PA and fruit or vegetable consumption	PA and diet	Website	No intervention	8	Yes	CES-D (secondary)		No		4	
Duan et al [54], 2018	China	Individuals with coronary heart disease; 114; 57%	Efficacy for PA and fruit or vegetable consumption	PA and diet	Website	Treatment as usual	8	Yes	CES-D (secondary)		No		5	
Eberts et al [55], 2015	Germany	Teachers with insomnia symptoms; 128; 74%	Efficacy for work-related stress and sleep	Sleep	Website	Waitlist	8	Yes	CES-D (secondary)		No		7	
Eckert et al [56], 2022	United States	Individuals with allogenic bone marrow transplant; 72; 55%	Feasibility	PA	Website	Attention control	12	No	PROMIS^n^ (exploratory)		Yes; Udaya		7	
Edney et al [57], 2020	Australia	Community; 284; 74%	Effectiveness for PA	PA	App	Waitlist	12	Yes	DASS-21 (secondary)		No		6	
Espie et al [58], 2019	United Kingdom	Individuals experiencing insomnia; 1712; 78%	Effectiveness for health and well-being	Sleep	App	Treatment as usual	12	Yes	WEMWBS (primary), PHQ-9, and GAD-7^o^ (secondary)		Yes; Sleepio		8	
Falk et al [59], 2022	United States	Sedentary employees; 47; 74%	Associations with well-being, stress, mood, and fatigue	PA	Website	Waitlist	12	Yes	PSS^p^ (exploratory)		No		7	
Felder et al [60], 2020	United States	Pregnant women; 208; 100%	Efficacy for insomnia	Sleep	App	Treatment as usual	10	Yes	EPDS and GAD-7 (secondary)		Yes; Sleepio		7	
Freeman et al [61], 2017	United Kingdom	University students with insomnia; 3755; 71%	Effectiveness for insomnia, paranoia, and hallucinations	Sleep	App	Treatment as usual	10	Yes	PHQ-9, GAD-7, and WEMWBS (secondary)		Yes; Sleepio		10	
Glozier et al [62], 2019	Australia	Older men experiencing depression; 87; 0%	Effectiveness for depression and insomnia	Sleep	App	Attention control	12	Yes	CES-D (primary)		Yes; SHUTi		9	
Golsteijn et al [63], 2018	Netherlands	Patients with cancer; 478; 13%	Efficacy for PA	PA	Web based and wearable	Treatment as usual	12	Yes	HADS (secondary)		No		7	
Hershner and O’Brien [64], 2018	United States	College students; 551; 57%	Effectiveness for sleep behavior	Sleep	Web based	No intervention	8	No	PHQ-9 (secondary)		No		4	
Hilmarsdottir et al [65], 2020	Iceland	Individuals with type 2 diabetes; 37; 51%	Effectiveness for metabolic health	PA and diet	App	Not reported	52	Yes	HADS and PAID^q^ (exploratory)		Yes; SidekickHealth		7	
Horsch et al [66], 2017	Netherlands	Individuals experiencing insomnia; 151; 63%	Efficacy for insomnia	Sleep	App	Waitlist	7	Yes	HADS (secondary)		Yes; Sleepcare		8	
Houchen-Wolloff et al [67], 2018	United Kingdom	Individuals with coronary heart disease; 60; 10%	Feasibility of web-based cardiac rehab	PA	Website	Treatment as usual	8	Yes	HADS (exploratory)		Yes; ActivateYourHeart		8	
Huberty et al [68], 2019	United States	Individuals with myoloproliferative neoplasm; 62; 94%	Feasibility and preliminary effectiveness for QoL	PA	Website	Waitlist	12	No	PROMIS (exploratory)		No		8	
Ifejika et al [69], 2020	United States	Individuals who experienced stroke; 36; 44%	Feasibility and preliminary effectiveness for monitoring diet	Diet	App	Attention control	26	Yes	PHQ-9 (secondary)		Yes; Lose it!		6	
Joutsenniemi et al [70], 2014^r^	Finland	General population; 3274; 83%	Effectiveness of PA versus positive psychology on happiness	PA	Email	Attention control	16	No	BDI (secondary)		No		7	
Katz et al [71], 2018	United States	Individuals with rheumatoid arthritis; 96; 88%	Effectiveness for PA and fatigue	PA	Wearable	Treatment as usual	21	Yes	PHQ-8 (secondary)		No		7	
Kuhn et al [72], 2022	United States	Veterans; 50; 42%	Feasibility, acceptability, and preliminary efficacy for insomnia	Sleep	App	Waitlist	6	Yes	PHQ-8 and GAD-7 (secondary)		Yes; insomnia coach		9	
Lambert et al [73], 2018	United Kingdom	Individuals who experienced depression; 62; 84%	Feasibility and acceptability in community setting	PA	Web based	Treatment as usual	8	Yes	PHQ-8 and GAD-7 (secondary)		No		6	
Lambert et al [74], 2022	Canada	Individuals with prostate cancer; 49; 0%	Feasibility, acceptability, and clinical significance for anxiety and QoL	PA	Web based	Waitlist	10	Yes	HADS (secondary)		No		7	
Lopez et al [75], 2019	France	Individuals experiencing insomnia; 46; 74%	Efficacy for insomnia	Sleep	Web based	Treatment as usual	12	Yes	BDI (secondary)		No		7	
Lorenz et al [76], 2019	Switzerland	Individuals experiencing insomnia; 56; 69%	Efficacy for insomnia	Sleep	Web based	Waitlist	6	Yes	BDI and APSQ^s^ (secondary)		Yes; Momentor somnium		9	
McGrath et al [77], 2017	United Kingdom	Individuals with elevated blood pressure; 134; 61%	Efficacy for reducing blood pressure	Sleep	Web based and text or email	Treatment as usual	8	Yes	BDI and BAI^t^ (secondary)		Yes; Sleepio		9	
Mensorio [78], 2019	Spain	Individuals with obesity and hypertension; 106; 80%	Efficacy for promoting lifestyle changes	PA and diet	Web based	Treatment as usual	12	Yes	DASS-21 (secondary)		No		7	
Mueller et al [79], 2022	United Kingdom	Individuals who are overweight or obese; 388; 78%	Effectiveness for bodyweight, eating behavior, PA, and well-being	PA and diet	Web based and email	Treatment as usual	12	Yes	PHQ-9 and GAD-7 (secondary)		No		8	
Murawski et al [80], 2019	Australia	Individuals who are inactive and experience poor sleep; 160; 80%	Efficacy for sleep and PA	PA and sleep	App, wearable, and email or text	Waitlist	12	Yes	DASS-21 (secondary)		Yes; balanced		6	
Nystrom et al [81], 2017	Sweden	Individuals experiencing depression; 312; 76%	Evaluate or compare PA and behavior activation	PA	App and text or email	Waitlist	12	Yes	PHQ-9 and GAD-7 (secondary)		No		7	
Philippot et al [82], 2022	Belgium	Higher education students; 30; 92%	Feasibility for psychological symptoms	PA	Web based	No intervention	4	No	DASS-21 (secondary)		No		7	
Przybylko et al [83], 2021	Australia	General population; 508; 70%	Effectiveness for mental health and well-being	PA and diet	Web based	Waitlist	10	Yes	DASS-21 (primary)		Yes; The Lift Project		6	
Puig-Ribera et al [84], 2017	Spain	Office workers; 264; 65%	Effectiveness for presenteeism, well-being, and performance	PA	Web based and wearable	Attention control	19	Yes	WEMWBS (secondary)		No		6	
Puterman et al [85], 2022	Canada	Inactive general population; 334; 87%	Effectiveness for depressive symptoms	PA	App	Waitlist	6	No	CES-D (primary)		No		6	
Ritterband et al [86], 2012	United States	Patients with cancer experiencing insomnia; 28; 86%	Effectiveness for insomnia	Sleep	Web based and email	Waitlist	9	No	HADS (secondary)		Yes; SHUT-i		8	
Savard et al [87], 2014^r^	Canada	Individuals with breast cancer; 242; 100%	Efficacy of video-based cognitive behavioral therapy in breast cancer	Sleep	DVD	No intervention	6	Yes	HADS (secondary)		No		10	
Serrat et al [88], 2022	Spain	Individuals with fibromyalgia; 330; 97%	Effectiveness for fibromyalgia	PA	Web based	Treatment as usual	12	No	HADS (secondary)		No		7	
Shaffer et al [89], 2022	United States	Older adults; 311; 69%	Effectiveness for depression and anxiety	Sleep	Web based and email	Attention control	10	Yes	HADS (primary)		Yes; SHUT-i		3	
Spanhel et al [90], 2022	Germany	Refugees; 66; 27%	Feasibility for insomnia and sleep	Sleep	Web based	Waitlist	4	Yes	PHQ-9 (secondary)		No		8	
Stiglbauer et al [91], 2019	Austria	University students; 105; 63%	Efficacy of self-tracking device on health	PA	App and wearable	Waitlist	2	Yes	PERMA^u^-Profiler (secondary)		No		5	
Teychenne et al [92], 2021	Australia	Postpartum women; 62; 100%	Feasibility and acceptability for health behaviors	PA	Web based	No intervention	12	Yes	EPDS and GAD-7 (secondary)		No		7	
Vandelanotte et al [93], 2022	Australia	General population; 501; 72%	Efficacy on depression, anxiety, stress, and QoL	PA	Web based	Treatment as usual	12	Yes	DASS-21 (primary)		No		6	
Wan et al [94], 2017	United States	Individuals with coronary obstructive pulmonary disease; 118; 2%	Effectiveness for increasing step count	PA	Web based and wearable	Attention control	12	Yes	BDI (secondary)		No		8	
Wang et al [95], 2022	China	Individuals with PCOS^v^; 122; 100%	Effectiveness for PCOS	PA and diet	App	Treatment as usual	52	Yes	SDS^w^ and SAS^x^ (secondary)		No		7	
Wang F and Boros S [96], 2020	Hungary	Young adults; 54; 53%	Effectiveness for sleep quality, stress, and life satisfaction	PA	Wearable	No intervention	4	Yes	PSS-4 and SWLS^y^ (secondary)		No		5	
Wong et al [97], 2021	China	Individuals with depressive symptoms; 79; 85%	Efficacy for depressive symptoms	PA, diet, and sleep	App	Waitlist	8	Yes	PHQ-9 and GAD-7 (primary)		No		7	
Young et al [98], 2021	Australia	Individuals who are overweight or obese; 125; 0%	Effectiveness for weight loss and depression	PA, diet, and sleep	Web based and wearable	No intervention	12	Yes	PHQ-9 (primary) and GAD-7 (secondary)		Yes; SHED-IT		7	
Xuto et al [99], 2022	Thailand	Pregnant women; 66; 100%	Effectiveness for health behaviors and anxiety	PA and diet	SMS text messages	Treatment as usual	28	No	STAI^z^ (secondary)		No		8	
Yudi et al [100], 2017	Australia	Individuals with acute coronary syndrome; 2016; 16%	Efficacy for exercise capacity and cardiac risk factor	PA	App	Treatment as usual	8	Yes	CDS^aa^ and HADS-A (secondary)		No		8	

^a^PEDro: Physiotherapy Evidence Database Scale.

^b^BCT: behavior change technique.

^c^PA: physical activity.

^d^PHQ-9: 9-item Patient Health Questionnaire.

^e^BDI: Beck Depression Inventory.

^f^QoL: Quality of life.

^g^WEMWBS: Warwick-Edinburgh Mental Well-Being Scale.

^h^EPDS: Edinburgh postnatal depression scale.

^i^OA: osteoarthritis.

^j^HADS: Hospital Anxiety and Depression Scale.

^k^DASS: Depression Anxiety and Stress Scale.

^l^SLS: selective laser sintering.

^m^CES-D: Centre for Epidemiological Studies Depression.

^n^PROMIS: Patient-Reported Outcomes Measurement Information System.

^o^GAD-7: Generalized Anxiety Disorder-7.

^p^PSS: Perceived Stress Scale.

^q^PAID: problem areas in diabetes.

^r^Not included in quantitative analyses.

^s^APSQ: Perceived Stress Scale.

^t^BAI: bay-annulated indigo.

^u^PERMA: Positive Emotions, Engagement, Relationships, Meaning, Accomplishment.

^v^PCOS: Polycystic Ovarian Syndrome.

^w^SDS: Self-Rating Depression Scale.

^x^SAS: Self-Rating Anxiety Scale.

^y^SWLS: Satisfaction with Life Scale.

^z^STAI: State Trait Anxiety Inventory.

^aa^CDS: Cardiac Depression Scale.

A total of 55 studies assessed depressive symptoms [40-42,44-58,60-83,85-90,92-95,97,98,100], 35 assessed anxiety symptoms [45,46,50-52,57,58,60,61,63,65-68,72-74, 76-83,86,88,89,92,93,95,97-100], 11 assessed stress [44,46,57,59,65,78,80,82,83,93,96], and 6 assessed well-being [43,46,61,84,91,96]. Overall, 24 studies included apparently healthy adults [44,46,48,50,51,53,55,57,59,64,70, 76,80,82-85,89-93,99]; 23 included adults with a physical health condition [43,45,47,52,54,56,63,65,67-69,71,74,77-79,86, 88,94,95,98,100]; 6 included adults with a mental health condition, all of which were depressive disorders [40,41,62,73,81,97]; and a further 8 included adults with insomnia [49,58,60,61,66,72,75,87]. Interventions were digitally delivered via the web (38/61, 62%) [40,44,45,48-50,52-56,58-64,67,68,73-79,82-84,86,88-90,92-94,98], app (17/61, 28%) [42,43,46,47,57,60,65,66,69,72,80,81, 85,91,95,97,100], email, or SMS text messages (15/61, 25%) [44,46,51,58,61,63,70,77,79-81,86,89,99,100], and 16% (10/61) of the interventions included a wearable activity tracker [41,42,57,63,71,80,91,94,96,98]. A total of 15 interventions used >1 delivery method [42,44,57,58,61,63,77,79-81,86,89,91,94,98]. Of the 61 interventions, 26 (43%) targeted physical activity [41,42,45,48,56,57,59,63,67,68,70,71,73,74,81,82,84,85, 88,91-94,96,100], 17 (29%) targeted sleep [49,50,55,58,60-62,64,66,72,75-77,86,87,89,90], 2 (3%) targeted diet [51,69], and 16 (27%) targeted multiple lifestyle behaviors [42,44,57,58,61,63,79-81,86,89,91,94,98]. Of the 17 studies targeting multiple behaviors, 4 (24%) targeted physical activity, diet, and sleep [40,47,97,98]; 11 (65%) targeted physical activity and diet [44,46,52-54,65,78,79,83,95,99]; and 2 (12%) targeted physical activity and sleep [43,80].

Mental health (ie, depression, anxiety, stress, and well-being) outcomes were measured as a primary outcome in 14 studies [41,46-51,62,83,85,89,93,97,98]. The risk of bias of the included studies is detailed in Multimedia Appendix 4.

### Meta-Analyses

For 2 of the included studies, the outcome data of interest was not presented in the manuscript and was unavailable after contacting the corresponding author [70,87].

#### Publication Bias

Visual inspection of funnel plots (Multimedia Appendix 5) suggests that there was no publication bias for depression (Kendall τ=−0.093, *P*=.33; Egger regression=0.19, *P*=.85), anxiety (Kendall τ=−0.106, *P*=.38; Egger regression=0.90, *P*=.37), or stress (Kendall τ=−0.055, *P*=.88; Egger regression=−0.11, *P*=.92).

#### Heterogeneity

There was significant heterogeneity across study data for depression (*Q*=166; *P*<.001; *I*^2^=69%) and anxiety (*Q*=55; *P*=.01; *I*^2^=39%). Stress and well-being data had moderate, nonsignificant heterogeneity (*Q*=17; *P*=.07; *I*^2^=42% and *Q*=8; *P*=.15; *I*^2^=39%, respectively).

### Overall Effects of Digital Lifestyle Interventions on Symptoms of Depression

**Figure 2:**
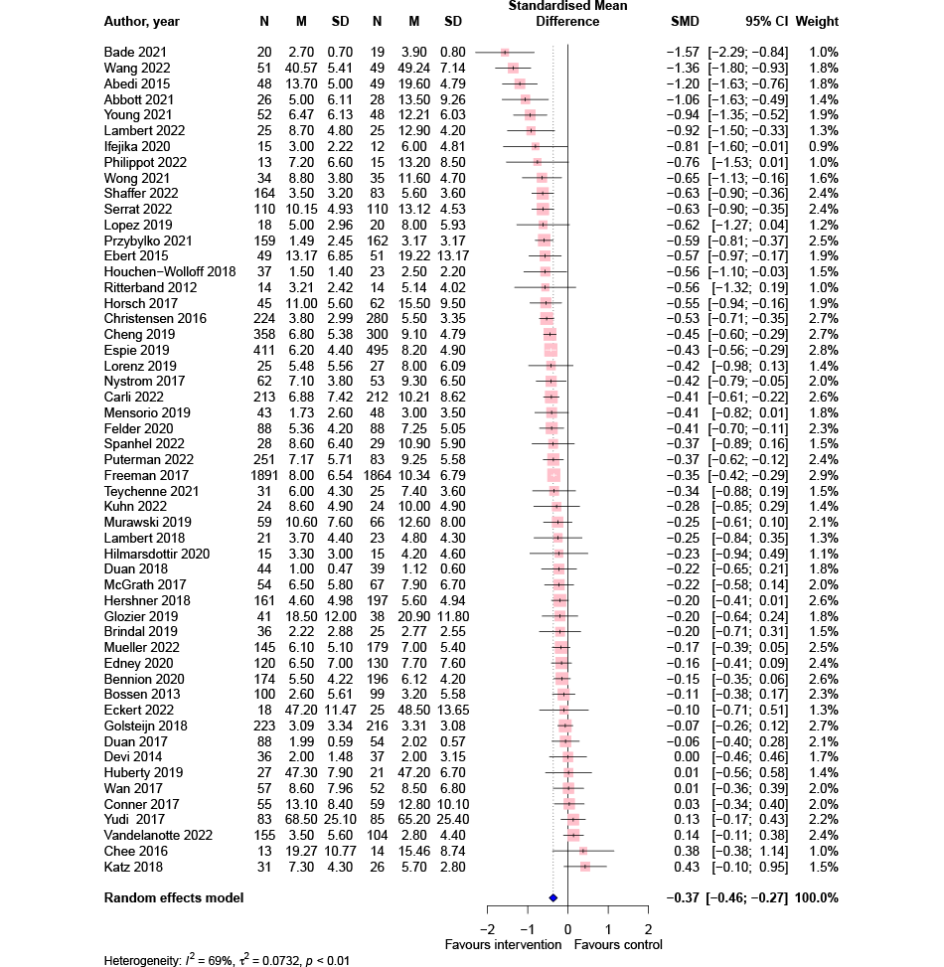
Meta-analysis of the effects of digital lifestyle interventions on symptoms of depression [40-42,44-58,60-69,71-83,85,86,88-90,92-95,97,98,100]. SMD: standardized mean difference.

Pooled effects from 53 studies (ie, 12,385 participants) showed a small-to-medium significant effect of digital lifestyle interventions for reducing depressive symptoms in comparison to all control conditions (SMD=−0.37; 95% CI −0.46 to −0.27; *P*<.001; Figure 2).

### Overall Effects of Digital Lifestyle Interventions on Symptoms of Anxiety

**Figure 3:**
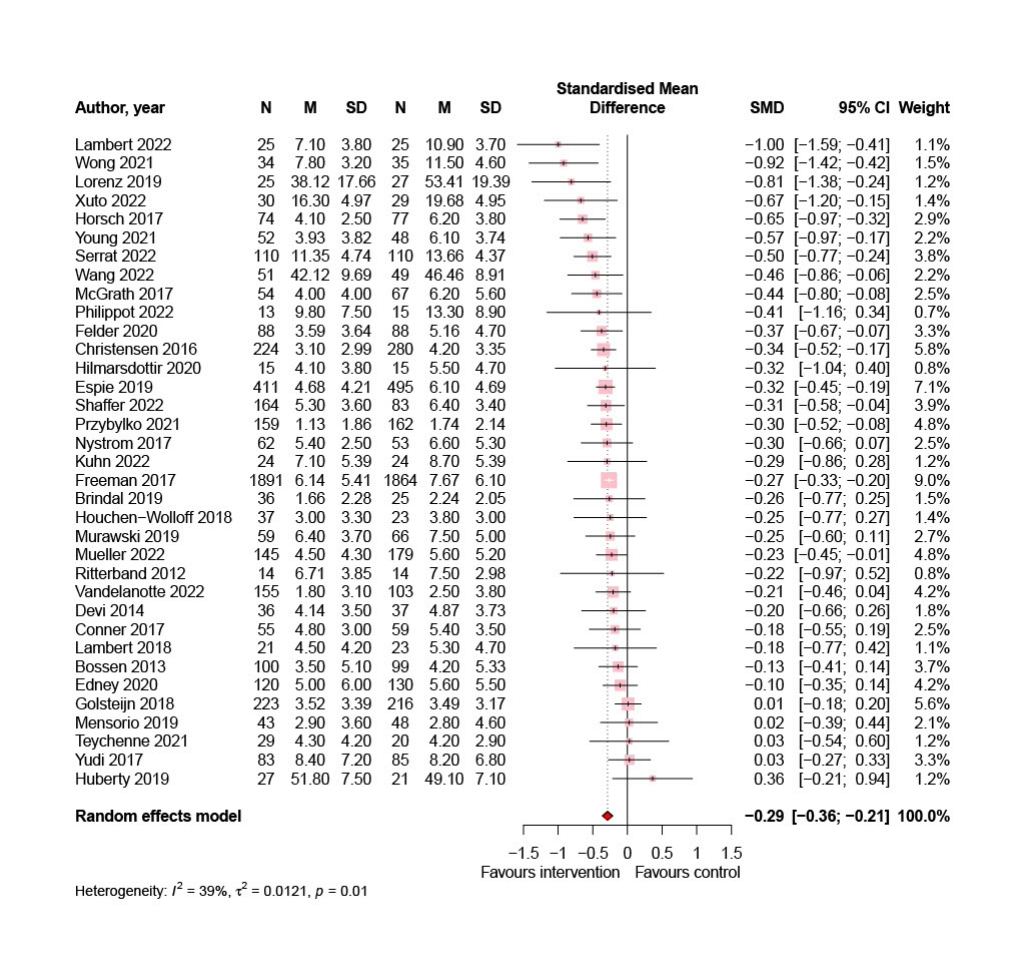
Meta-analysis of the effects of digital lifestyle interventions on symptoms of anxiety [45,46,50-52,57,58,60,61,63,65-68,72,73,75-83,86,88,89,92,93,95,97-100]. SMD: standardized mean difference.

Figure 3 displays the pooled effect size from 35 studies (9383/22,483, 41.73%), showing a small-to-medium positive effect of digital lifestyle interventions compared to all control conditions on symptoms of anxiety (SMD=−0.29; 95% CI −0.36 to −0.21; *P*<.001).

### Overall Effects of Digital Lifestyle Interventions on Stress

**Figure 4:**
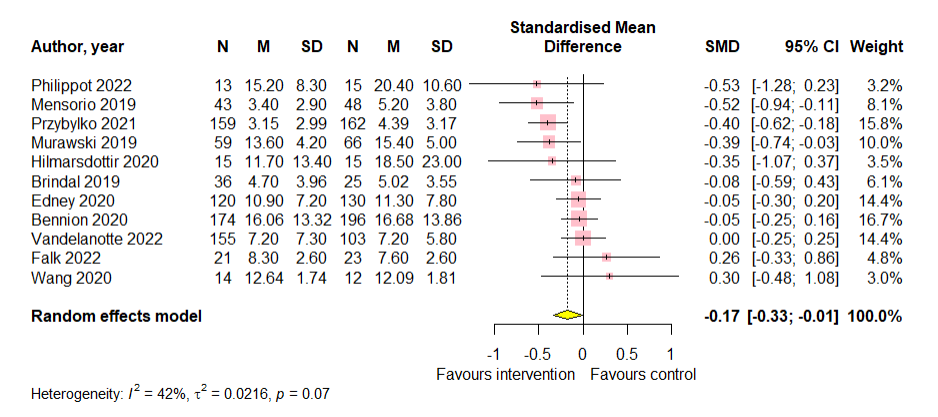
Meta-analysis of the effects of digital lifestyle interventions on stress [44,46,57,59,65,78,80,82,83,93,96]. SMD: standardized mean difference.

Pooled effects from 11 studies (n=1608) showed a small positive effect of digital lifestyle interventions compared to all control conditions on stress (SMD=−0.17; 95% CI −0.33 to −0.01; *P*=.04; Figure 4).

### Overall Effects of Digital Lifestyle Interventions on Well-Being

**Figure 5:**
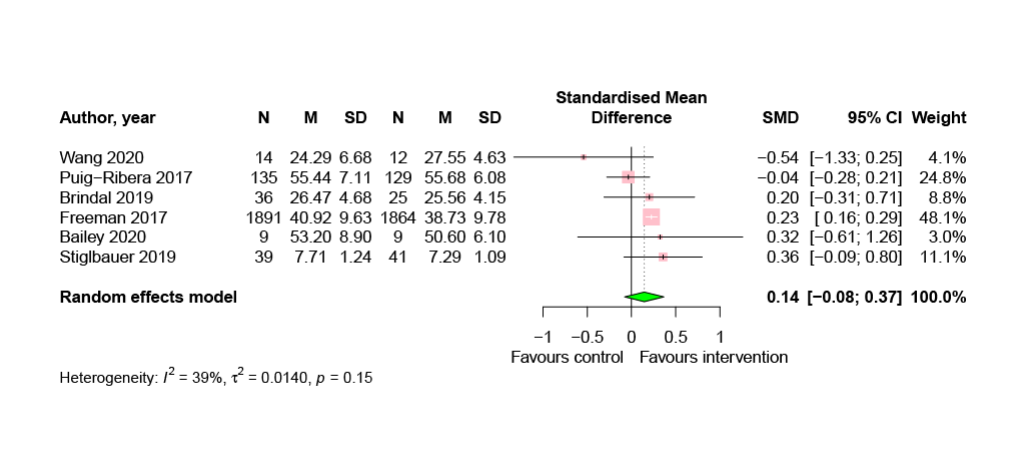
Meta-analysis of the effects of digital lifestyle interventions on well-being [43,46,61,84,91,96]. SMD: standardized mean difference.

Pooled effects from 6 studies (4204/22,483, 18.7%) suggest no significant effect of digital lifestyle interventions compared to all control conditions on well-being (SMD=0.14; 95% CI −0.07 to 0.37; *P*=.15; Figure 5), although the data show a nonsignificant (*P*=.15) trend in the direction that favors digital lifestyle interventions.

### Subgroup Analyses

#### Overview

To understand which aspects of digital lifestyle interventions contribute to their effectiveness in improving mental health and well-being, and for whom they are most beneficial, we conducted a series of comparative subgroup analyses. These analyses were based on technology-specific features, methodological approaches, intervention characteristics, and population characteristics. Due to the limited number of studies for stress and well-being, these analyses were conducted for depressive and anxiety outcomes only. The results are outlined in Tables 2 and 3.

**Table 2:** Subgroup analyses of depression outcomes.

					Number of studies, k		Intervention, n		Control, n		Meta-analysis					Heterogeneity					Subgroup test, *P* value
											Standardized mean difference (95% CI)		*P* value		*Q*			*I*^2^ (%)			
Overall					53		6285		6100		−0.37 (−0.4641 to −0.2673)		<.0001		166.32			68.7		—^a^	
**Population characteristics**																					
	General population				17		1792		1586		−0.30 (−0.4252 to −0.1753)		<.001		43.41			63.1		.46	
	Mental diagnosis				7		288		288		−0.51 (−0.9005 to −0.1131)		.02		21.72			67.8		—	
	Physical diagnosis				21		1370		1373		−0.36 (−0.5828 to −0.1455)		<.001		87.88			77.2		—	
	Insomnia				7		2835		2853		−0.39 (−0.4428 to −0.3350)		<.001		3.40			3.4		—	
**Intervention characteristics**																					
	**Behavior**																				
		Diet		2		70		71		−0.31 (−5.5627 to 4.9394)		.59		3.55			71.8		.75		
		Physical activity		20		1445		1197		−0.29 (−0.5138 to −0.0708)		.01		79.58			76.1		—		
		Sleep		16		3595		3639		−0.41 (−0.4662 to −0.3437)		<.001		14.58			0		—		
		Multiple behavior		15		1175		1193		−0.43 (−0.6452 to −0.2173)		<.001		52.71			73.4		—		
	**Frequency**																				
		Daily		17		1336		1368		−0.32 (−0.5658 to −0.0695)		.02		69.12			76.9		.40		
		1-4 times per wk		26		1880		1598		−0.34 (−0.4473 to −0.2368)		<.001		53.52			53.3		—		
		Other^b^		10		3069		3134		−0.52 (−0.8015 to −0.2339)		<.001		42.91			79		—		
	**Duration**																				
		≤6 wk		9		682		582		−0.38 (−0.6239 to −0.1410)		.006		16.47			51.4		.95		
		7-16 wk		37		5244		5172		−0.35 (−0.4639 to −0.2454)		<.001		113.61			68.3		—		
		≥4 mo		7		359		346		−0.40 (−0.9378 to 0.1295)		.11		34.87			82.8		—		
	**Individualized**																				
		No		16		1003		1054		−0.40 (−0.6365 to −0.1716)		<.001		64.61			76.8		.64		
		Yes		37		5282		5046		−0.35 (−0.4538 to −0.2400)		<.001		101.5			64.5		—		
	**Theoretical underpinning**																				
		No		31		4715		4603		−0.39 (−0.4850 to −0.2945)		<.001		60.33			50.3		.69		
		Yes		22		1570		1497		−0.35 (−0.5497 to −0.1444)		<.001		69.35			78.2		—		
	**Co-designed**																				
		No		47		5927		5707		−0.38 (−0.4864 to −0.2714)		<.001		154.81			70.3		.09		
		Yes		6		358		393		−0.20 (−0.4351 to 0.0271)		0.07		7.47			33.1		—		
	**Publicly available**																				
		No		32		2330		2147		−0.35 (−0.5054 to −0.1878)		<.001		123.4			74.9		.42		
		Yes		21		3955		3953		−0.42 (−0.4961 to −0.3381)		<.001		29.88			33.1		—		
	**Delivery method**																				
		**Wearable**																			
			No	45		5675		5494		−0.36 (−0.4473 to −0.2698)		<.001		110.04			60		.74		
			Yes	8		610		606		−0.44 (−0.9897 to 0.1122)		.10		53.74			87		—		
		**App**																			
			No	38		5169		5142		−0.33 (−0.4450 to −0.2248)		<.001		117.66			68.6		.31		
			Yes	15		1116		958		−0.46 (−0.6866 to −0.2263)		<.001		47.98			70.8		—		
		**Web based**																			
			No	17		1162		1004		−0.44 (−0.6971 to −0.1767)		<.001		75.3			78.8		.46		
			Yes	36		5123		5096		−0.34 (−0.4329 to −0.2451)		<.001		90.78			61.4		—		
		**Text or email**																			
			No	40		2930		2704		−0.39 (−0.5114 to −0.2767)		<.001		118.39			67.1		.31		
			Yes	13		3355		3396		−0.29 (−0.4770 to −0.0945)		<.001		44.25			72.9		—		
		**Multiple methods**																			
			No	39		2839		2618		−0.37 (−0.4907 to−0.2499)		<.001		119.97			68.3		.82		
			Yes	14		3446		3482		−0.35 (−0.5333 to −0.1598)		<.001		45.17			71.2		—		
	**Delivery features**																				
		**Video**																			
			No	36		2494		2537		−0.41 (−0.5435 to −0.2760)		<.001		112.77			69		.24		
			Yes	17		3791		3563		−0.30 (−0.4402 to −0.1596)		<.001		53.10			69.9		—		
		**Chatbot**																			
			No	47		5134		4848		−0.35 (−0.4665 to −0.2397)		<.001		156.18			70.5		.08		
			Yes	6		1151		1252		−0.46 (−0.5112 to −0.4055)		<.001		1.22			0		—		
		**Gamification or interaction**																			
			No	40		3193		3031		−0.36 (−0.4785 to −0.2362)		<.001		129.65			69.9		.69		
			Yes	13		3092		3069		−0.40 (−0.5797 to −0.2176)		<.001		34.28			65		—		
		**Notifications**																			
			No	32		4102		3898		−0.33 (−0.4756 to −0.1915)		<.001		109.38			71.7		.45		
			Yes	21		2183		2202		−0.41 (−0.5399 to −0.2705)		<.001		54.74			63.5		—		
		**Social features**																			
			No	37		5149		4961		−0.36 (−0.4726 to −0.2525)		<.001		105.75			66		.96		
			Yes	16		1136		1139		−0.37 (−0.5950 to −0.1426)		<.001		60.26			75.1		—		
**Methodological characteristics**																					
	**Control group**																				
		Waitlist control or none		36		4135		3988		−0.44 (−0.5629 to −0.3111)		<.001		101.68			65.6		.11		
		Treatment as usual		11		1626		1522		−0.22 (−0.4330 to −0.0081)		.04		47.08			78.8		—		
		Attention		6		524		590		−0.27 (−0.5498 to 0.0089)		.06		13.32			62.5		—		
	**Outcome measure**																				
		Primary		13		1751		1444		−0.39 (−0.6066 to −0.1666)		.002		50.31			76.1		.64		
		Secondary		36		4437		4572		−0.37 (−0.4934 to −0.2438)		<.001		108.51			67.7		—		
		Exploratory		4		97		84		−0.23 (−0.6696 to 0.1958)		0.18		2.40			0		—		
	**Study quality**																				
		Poor to fair		5		470		387		−0.23 (−0.6161 to 0.1573)		.17		11.44			65.0		.53		
		Good		41		3198		3113		−0.39 (−0.5169 to −0.2660)		<.001		146.70			72.7		—		
		Excellent		7		2617		2600		−0.39 (−0.4768 to −0.3020)		<.001		5.79			0		—		
	**Publication year**																				
		2014 or earlier		3		150		150		−0.12 (−0.5666 to 0.3230)		.36		1.59			0		.01		
		2015-2017		12		2669		2690		−0.29 (−0.5450 to −0.0515)		.02		73.81			77.0		—		
		2018-2020		20		1947		2005		−0.27 (−0.3636 to −0.1753)		<.001		45.06			75.6		—		
		2021or later		18		1519		1255		−0.57 (−0.7795 to −0.3635)		<.001		30.47			37.7		—		

^a^Not applicable.

^b^Other frequency includes fortnightly, monthly, once-off, and self-paced.

**Table 3:** Subgroup analyses of anxiety outcomes.

						Number of studies, k			Intervention, n			Control, n			Meta-analysis						Heterogeneity							Subgroup test, *P* value
															Standardized mean difference (95% CI)			*P* value			*Q*			*I*^2^ (%)				
Overall						35			4689			4694			−0.29 (−0.3620 to −0.2116)			<.001			55.42			38.7			—^a^	
**Population**																												
	General population				11			905			916			−0.28 (−0.3829 to −0.1731)			<.001			9.84			0			.94		
	Mental diagnosis				4			160			159			−0.33 (−0.9751 to 0.3087)			.19			8.52			64.8			—		
	Physical diagnosis				14			972			988			−0.26 (−0.4282 to −0.0940)			<.001			29.52			56.0			—		
	Insomnia				6			2652			2631			−0.30 (−0.3947 to −0.2113)			<.001			5.60			10.7			—		
**Intervention characteristics**																												
	**Behavior**																											
		Diet		1			55			59			−0.18 (−0.5505 to 0.1858)			.33			0			0			.47			
		Physical activity		14			1035			952			−0.20 (−0.3696 to −0.0388)			.01			27.28			52.4			—			
		Sleep		10			2969			3019			−0.33 (−0.4024 to −0.2486)			<.001			9.68			7.0			—			
		Multi		10			630			664			−0.31 (−0.4536 to −0.1672)			<.001			11.41			21.1			—			
	**Frequency**																											
		Daily		8			631			695			−0.26 (−0.4352 to −0.0776)			.01			11.44			38.8			.65			
		Weekly		19			1236			1204			−0.35 (−0.4289 to −0.2175)			<.001			25.36			29.0			—			
		Other^b^		8			2822			2795			−0.25 (−0.4396 to −0.0653)			.02			16.81			58.3			—			
	**Duration**																											
		≤6 wk		6			377			442			−0.34 (−0.4925 to −0.1771)			.003			3.76			0			.31			
		7-16 wk		24			4143			4111			−0.27 (−0.3705 to −0.1700)			<.001			47.57			51.7			—			
		≥4 mo		5			169			141			−0.41 (−0.6219 to −0.1924)			.006			1.78			0			—			
	**Individualized**																											
		No		11			726			742			−0.28 (−0.5126 to −0.0560)			.02			26.41			62.1			.99			
		Yes		24			3963			3952			−0.28 (−0.3544 to −0.2140)			<.001			29.01			20.7			—			
	**Theoretical underpinning**																											
		No		21			3594			3657			−0.29 (−0.3499 to −0.2390)			<.001			23.45			14.7			.73			
		Yes		14			1095			1037			−0.26 (−0.4315 to −0.1010)			.004			29.12			55.4			—			
	**Co-designed**																											
		No		31			4383			4354			−0.29 (−0.3689 to −0.2100)			<.001			48.41			38.0			.93			
		Yes		4			306			340			−0.31 (−0.8339 to 0.2240)			0.16			6.91			56.6			—			
	**Publicly available**																											
		No		19			1350			1313			−0.25 (−0.3946 to −0.1114)			.001			39.57			54.5			0.51			
		Yes		16			3339			3381			−0.31 (−0.3519 to −0.2486)			<.001			12.86			0			—			
	**Delivery methods**																											
		**Wearable**																										
			No	31			4235			4234			−0.29 (−0.3450 to −0.2374)			<.001			42.14			28.8			.36			
			Yes	4			454			460			−0.18 (−0.5553 to 0.1952)			0.22			7.18			58.2			—			
		**App**																										
			No	24			4043			4047			−0.27 (−0.3562 to −0.1905)			<.001			36.93			37.7			.52			
			Yes	11			646			647			−0.33 (−0.5017 to −0.1572)			.002			18.19			45.0			—			
		**Web based**																										
			No	12			643			647			−0.33 (−0.5043 to −0.1623)			.001			20.29			45.8			.49			
			Yes	23			4046			4047			−0.27 (−0.3548 to −0.1922)			<.001			34.91			37.0			—			
		**Text or email**																										
			No	23			1498			1484			−0.33 (−0.4464 to −0.2169)			<.001			36.98			40.5			.17			
			Yes	12			3191			3210			−0.24 (−0.3309 to −0.1421)			<.001			15.81			30.4			—			
		**Multiple methods used**																										
			No	24			1494			1479			−0.32 (−0.4375 to −0.2033)			<.001			39.94			42.4			.29			
			Yes	11			3195			3215			−0.25 (−0.3375 to −0.1562)			<.001			13.68			26.9			—			
	**Delivery features**																											
		**Video**																										
			No	25			1810			1840			−0.30 (−0.3823 to −0.2206)			<.001			28.87			16.9			.63			
			Yes	10			2879			2854			−0.25 (−0.4528 to −0.0569)			.02			25.61			64.9			—			
		**Chatbot**																										
			No	30			3867			3727			−0.25 (−0.3373 to −0.1717)			<.001			44.6			35.0			.10			
			Yes	5			822			967			−0.37 (−0.5304 to −0.2107)			.003			5.78			30.7			—			
		**Gamification or interaction**																										
			No	23			1610			1639			−0.27 (−0.3771 to −0.1705)			<.001			36.56			39.8			.91			
			Yes	12			3079			3055			−0.28 (−0.3546 to −0.2070)			<.001			18.64			41.0			—			
		**Notifications**																										
			No	19			3072			3034			−0.23 (−0.3652 to −0.1042)			.001			37.21			51.6			.15			
			Yes	16			1617			1660			−0.34 (−0.4087 to −0.2658)			<.001			13.33			0			—			
		**Social features**																										
			No	25			3957			3943			−0.29 (−0.4038 to −0.1912)			<.001			48.83			50.8			.70			
			Yes	10			732			751			−0.27 (−0.3727 to −0.1698)			<.001			6.59			0			—			
**Methodological characteristics**																												
	**Control group**																											
		Waitlist control or none		25			3221			3223			−0.31 (−0.4068 to −0.2061)			<.001			37.88			36.6			.46			
		Treatment as usual		8			1208			1166			−0.24 (−0.4126 to −0.0741)			.01			16.01			56.3			—			
		Attention		2			260			305			−0.34 (−0.6574 to −0.0126)			.05			0.09			0			—			
	**Outcome measure**																											
		Primary		7			827			747			−0.32 (−0.4533 to −0.1792)			.001			7.09			15.3			.50			
		Secondary		25			3783			3888			−0.29 (−0.3877 to −0.1973)			<.001			42.9			44.1			—			
		Exploratory		3			79			59			−0.05 (−0.9823 to 0.8771)			.83			3.06			34.6			—			
	**Study quality**																											
		Poor		1			164			83			−0.31 (−0.5757 to −0.0448)			.02			0			0			.87			
		Good		29			2307			2349			−0.27 (−0.3684 to −0.1805)			<.001			50.24			44.3			—			
		Excellent		5			2218			2262			−0.31 (−0.4406 to −0.1751)			.003			4.79			16.5			—			
	**Publication year**																											
		2014 or earlier		3			150			150			−0.16 (−0.2635 to −0.0525)			.02			0.09			0			.003			
		2015-2017		7			2443			2485			−0.30 (−0.4578 to −0.1345)			.004			10.55			43.1			—			
		2018-2020		12			1105			1177			−0.19 (−0.3372 to −0.0500)			.01			19.17			42.6			—			
		2021 or later		13			991			882			−0.39 (−0.5282 to −0.2492)			<.001			18.21			34.1			—			

^a^Not applicable.

^b^Other frequency includes fortnightly, monthly, once-off, and self-paced.

#### Delivery Method and Features

The effectiveness of interventions for improving depression and anxiety did not significantly differ based on the digital delivery method used or their technological features (eg, video, gamification, notifications, interactive design, and social features). There was a nonsignificant trend for interventions using chatbots to be slightly more effective for reducing depressive symptoms than interventions not using chatbots (*P*=.08).

#### Intervention Characteristics

Interventions had similar effects on depression and anxiety, regardless of whether this was achieved through physical activity, sleep, diet, or multiple lifestyle behaviors. Diet-only interventions demonstrated nonsignificant effects on depression and anxiety, likely due to the paucity of studies.

Intervention effectiveness did not differ based on the frequency or duration of the intervention; however, long-term interventions (≥4 months) demonstrated a nonsignificant effect on depressive symptoms. Intervention effectiveness did not differ based on the intervention being personalized, based on a theoretical framework, whether they had been co-designed with consumers, or whether they were publicly available.

#### Methodological Characteristics

The effectiveness of interventions did not differ when comparing waitlist or no treatment controls, treatment-as-usual controls, and attention or sham controls. Similarly, effectiveness did not differ based on study quality.

Considering we included studies that did not specifically target mental health, but captured it as an outcome, we compared interventions with depression or anxiety as a primary outcome to those capturing these as secondary outcomes or exploratory outcomes. Intervention effectiveness did not differ based on whether the outcome was primary or secondary; however, the few studies that captured depression and anxiety as exploratory outcomes demonstrated nonsignificant intervention effects.

#### Population Characteristics

Digital lifestyle interventions demonstrated comparable effectiveness for improving depression and anxiety across all populations: apparently healthy adults, people with a mental disorder, people with a physical health condition, and people with insomnia. For depressive symptoms, the largest effect was seen in people with a mental disorder, whereas for anxiety symptoms, a nonsignificant effect was observed for those with a mental disorder.

#### Publication Year

The effectiveness of interventions on depression and anxiety differed significantly based on the year of publication, with more recently published interventions demonstrating the greatest effects.

Mixed-effects metaregression analysis showed no significant effect of intervention duration, study quality, or publication year on the effect of digital lifestyle interventions for depression, anxiety, stress, or well-being outcomes. A detailed overview of the metaregression analyses is provided in Multimedia Appendix 6.

### Sensitivity Analyses

Sensitivity analyses for all meta-analyses are detailed in Multimedia Appendix 7. For depression, when small studies were omitted from the analysis, the effect size shrunk but remained significant (SMD=−0.28; 95% CI −0.38 to −0.19; *P*<.001). For anxiety, the effect size was slightly larger when studies with >25% attrition were removed (SMD=−0.31; 95% CI −0.43 to −0.18; *P*<.001). For stress, omitting small studies or studies with >25% attrition no longer demonstrated a significant effect (*P*=.13 and *P*=.22, respectively). The overall effect on well-being remained nonsignificant when fair to poor quality studies, small studies, and studies with >25% attrition were removed (all *P* values >.10).

### Level of Evidence

Overall, the level of evidence for digital lifestyle interventions for improving symptoms of depression, anxiety, and stress is grade A: consistent level 1 studies. The grade of recommendation for digital lifestyle interventions for improving symptoms of well-being was grade D: level 5 evidence.

## Discussion

### Principal Findings

This systematic review aimed to comprehensively examine the effectiveness of digital lifestyle interventions for improving symptoms of depression, anxiety, stress, and well-being in adults. We identified 61 RCTs involving 22,483 participants. The findings suggest that digital lifestyle interventions had small-to-medium favorable effects on depression, anxiety, and stress symptoms across a broad range of populations and via a broad range of digital delivery methods. These effects are similar in magnitude to those observed for established mental health treatments, such as antidepressant medication [101] and psychotherapy [102]. No significant effect was observed for well-being, although in comparison to mental ill health, fewer studies (n=6) of lower average quality measured well-being outcomes. These studies operationalized well-being broadly using either global measures that span various affective and functional dimensions [43,61,84,91] or scales of overall life satisfaction [46,96]. These are broad constructs that tend to be relatively stable [103]. As a result, the available evidence offers limited precision for elucidating the effects of digital lifestyle interventions on specific well-being domains. Two-thirds of the included studies (41/61, 67%) were published within the last 5 years, reflecting the increased interest in using digital technology for health behavior change for mental health, with greater reductions in depressive and anxiety symptoms observed for more recently published interventions. This likely reflects the volume of studies published recently but may also be driven by improved quality of digital interventions as technology, particularly artificial intelligence, is better used within health promotion [104].

To better understand what factors drive the effects of digital lifestyle interventions on depressive and anxiety symptoms, we conducted a range of comparative subgroup analyses on specific features relating to the intervention, methodology, technology, and population. These analyses found that interventions demonstrated comparable effectiveness for reducing symptoms of depression and anxiety in apparently healthy adults or adults with a mental disorder, a physical health condition, or insomnia. Equivalent effectiveness was found regardless of the lifestyle behaviors targeted, method of digital delivery (eg, app and website), technological features (ie, chatbot and gamification), intervention duration, or intended frequency of engagement. Overall, our subgroup analyses indicated that there are no specific features that drive the effectiveness of digital lifestyle interventions for improving symptoms of depression or anxiety, except for publication year.

Although differences among population groups were not significant, it appears that digital lifestyle interventions are effective for improving depressive symptoms in people with a depressive disorder but are potentially less effective at improving symptoms of anxiety in people with a depressive disorder. Interestingly, depressive disorders were the only diagnosed mental disorder in the included studies, highlighting a clear gap. Therefore, the effectiveness of these interventions as a self-management approach for other mental disorders is unclear and should be explored in future studies.

We also observed that interventions using chatbots trended toward being more effective than those without chatbots, though these results were not statistically significant. These findings have been supported with recent research,. Emerging evidence suggests chatbots can help improve lifestyle behaviors [105], although it appears their effectiveness for delivering psychological interventions (ie, therapy) is less conclusive [106]. Co-designed interventions showed a trend for being less effective. This is likely due to the small number of studies included in the subgroup analysis and the high variation of co-design approaches used in the design of health interventions, which are often poorly described and rarely evaluated [107].

A key challenge of digital health interventions is maintaining user engagement over time [13]. It is possible that the lack of significant intervention effects at 4 months was due to drop off in engagement. Alternatively, it is possible that symptoms may recur in some individuals even if they remain engaged with the intervention, reflecting the recurring nature of mental health symptoms. Digital lifestyle interventions may offer an appropriate short-term strategy to engage people in mental health–promoting behaviors and initiate positive shifts in mental health, whereas more sustainable mental health benefits may require other mental health treatment approaches. These findings reinforce the existing literature that the clinical implications of lifestyle interventions, even when delivered digitally, are compelling as a self-management tool for targeting both the risk and protective factors for mental health and should be given consideration as part of standard mental health care. This review did not directly compare digital to face-to-face delivery of these interventions; therefore, the impact that delivery method has on effectiveness should be examined directly in future research. Given the role of patient-practitioner rapport and other psychosocial benefits of in-person care, it is pivotal to understand how digital interventions may best fit and integrate within the broader health care setting, including alongside gold standard treatments, to optimize patient outcomes.

In line with previous research [108], we found interventions targeting multiple or single behaviors to have comparable effectiveness. Interventions that aim to change multiple health behaviors concurrently seem logical, given the cooccurrence of and bidirectional relationship between unhealthy lifestyle behaviors and poor mental health. In particular, some health behaviors may have synergistic impacts (eg, people who improve their sleep may have more energy for physical activity and, in turn, people who are more physically active may sleep better); therefore, future research should explore how behavior change techniques can be implemented most effectively to address multiple behaviors and aim to identify moderators of effective interventions and whether delivering lifestyle behavior change within or alongside traditional psychological interventions has synergistic effects on outcomes or improves adherence.

This review also focused on adults, although adolescence is a period when mental health issues commonly present and offers an opportune time for preventive intervention. Digital interventions would seem to be appealing and accessible for this population, given their familiarity with technology. A recent review by Raeside et al [109] included 11 interventions and found small nonsignificant effects for digital lifestyle interventions on mental health outcomes in adolescents. Considering the shared protective and risk factors for mental health and chronic diseases, future research efforts should focus on if and how digital tools can be best harnessed to address the interplay between lifestyle behaviors, mental ill health, and well-being and engage young people in the design and development of interventions through participatory research methods, such as co-design.

### Strengths and Limitations

This is the largest review to evaluate the effects of digital interventions targeting lifestyle behaviors on depression, anxiety, stress, and well-being in adults. We adhered to rigorous methodological approaches for the conduct and reporting of systematic reviews and meta-analyses and conducted comprehensive database searches. In total, 87% (53/61) of the included RCTs received good or excellent quality ratings, increasing confidence in the findings of this review. Given the very large number of studies included in the meta-analyses, the dataset was sufficient to support numerous subgroup analyses, facilitating closer interrogation of the factors influencing intervention effectiveness. Our review included digital lifestyle interventions that have been evaluated using an RCT design and published in a peer-reviewed academic journal. Many commercial digital products claim to support lifestyle changes and mental health but often lack rigorous scientific assessment. The conclusions from this study should not be generalized to such unevaluated products.

Most of the study’s limitations arose from the limitations of the included studies. First, only 2 studies reported on diet-only interventions, and few studies measured stress and well-being, resulting in uncertain and underpowered results for these meta-analyses and precluding subgroup analyses with stress and well-being as outcomes. Second, all but 2 studies [41,99] were conducted in high-income countries, limiting the generalizability of our results. Third, it is important to acknowledge that our subgroup analyses, such as the comparison between single- and multibehavioral interventions, were conducted using available data from studies not specifically designed to test these differences. Consequently, these findings should be interpreted cautiously and should not be used as definitive evidence for or against any particular intervention approach. Finally, we acknowledge significant heterogeneity as a limitation, and we conducted numerous subgroup and sensitivity analyses to mitigate its impact and assess the robustness of our findings, which remained consistent with the main analyses.

### Future Directions

This review highlights some key areas for future research. First, there were limited studies focused on addressing diet as a stand-alone intervention; however, a nonsignificant effect in the direction of lifestyle interventions for reducing depressive symptoms highlights that further research is needed to better determine if, and how, effective these interventions may be in addressing mental health symptoms. Second, digital interventions may be less effective over longer periods, emphasizing a need to address longer-term strategies or the potential to integrate other mental health treatment approaches, which may demonstrate greater effects over time. Research also needs to address how best to integrate these evidence-based interventions into service delivery to support health care capacity. Third, for a more complete understanding of how digital lifestyle interventions influence mental health, future research should ensure that positive aspects of mental well-being are measured alongside symptoms of mental ill health. A growing body of literature suggests that these are distinct continua, with both overlapping and unique antecedents, that should be measured in tandem [110]. Finally, as the field continues to mature and research elucidates optimal intervention designs, other factors that are imperative to consider are the influence of both health literacy and digital literacy as well as the impact of health status and socioeconomic status [111]. Furthermore, future research should consider the digital determinants of health and the direct and indirect impacts on health equity when designing health promotion interventions [112].

### Conclusions

Overall, the small-to-medium effects indicate that digital lifestyle interventions may provide an effective short-term self-management strategy for mental health support by encouraging positive shifts in lifestyle behaviors. All digital delivery platforms and features demonstrated comparable effectiveness for depression and anxiety outcomes. The effectiveness on well-being is inconclusive, with few studies capturing this positive dimension of mental health. Digitally delivered lifestyle interventions support self-management and target both risk and protective factors for mental health and should be given consideration as part of mental health care and support. This is especially relevant in situations where access to in-person treatment is limited, such as due to long waitlists, financial or geographical barriers, or stigma associated with traditional treatment options.

Future research should explore how best to implement these findings and integrate such interventions with existing health care services, with concerted efforts to improve health equity by considering the digital determinants of health and impacts of digital and health literacy in the design and implementation of these interventions.

## Appendix

Multimedia Appendix 1 PRISMA checklist.

Multimedia Appendix 2 Search strategy.

Multimedia Appendix 3 Reasons for exclusion at full-text screening.

Multimedia Appendix 4 Physiotherapy Evidence Database Scale risk of bias for included studies.

Multimedia Appendix 5 Funnel plots for meta-analyses.

Multimedia Appendix 6 Meta-regression analyses for all outcomes.

Multimedia Appendix 7 Sensitivity analyses for all meta-analyses.

## Abbreviations

PEDro: Physiotherapy Evidence Database Scale
PRISMA: Preferred Reporting Items for Systematic Reviews and Meta-Analyses
RCT: randomized controlled trial
SMD: standardized mean difference

## Data Availability

The datasets generated or analyzed during this study are available from the corresponding author on reasonable request.
