# Supplementary material for: Effectiveness of Digital Lifestyle Interventions on Depression, Anxiety, Stress, and Well-Being: Systematic Review and Meta-Analysis
Source: J Med Internet Res. 2025 Mar 20;27:e56975. doi: 10.2196/56975 (PMC11969127; doi:10.2196/56975)
Supplement: Multimedia Appendix 2 [file jmir_v27i1e56975_app2.docx]

# Appendix 1.

## Search strategy for databases

### MEDLINE

| 1 | Internet-based interventions/ |
| --- | --- |
| 2 | Cell phone/ |
| 3 | Mobile applications/ |
| 4 | smartphone/ |
| 5 | Text Messaging/ |
| 6 | Social media/ |
| 7 | Fitness trackers/ |
| 8 | Wearable Electronic Devices/ |
| 9 | (internet-based intervention or web-based intervention or online intervention or mobile* or eHealth or mobile health or mHealth or digital health or online or email or website or digital intervention or mobile app* or smartphone or mobile phone or acceleromet* or pedomet* or wearable* or activity monitor* or remote or fitness tracker* or fitbit* or social media or social network or text messag* or chatbot or artificial intelligence or virtual assistant).ti,ab,kf. |
| 10 | 1 OR 2 OR 3 OR 4 OR 5 OR 6 OR 7 OR 8 OR 9 |
| 11 | Healthy lifestyle/ |
| 12 | Exercise/ |
| 13 | Sedentary Behavior/ |
| 14 | Sleep/ |
| 15 | Diet/ |
| 16 | (healthy eating or diet or weight loss or weight management or weight reduction program or physical activit* or exercis* or walk* or fitness or sedentar* behavior or sitting time or lifestyle).ti,ab,kf. |
| 17 | 11 OR 12 OR 13 OR 14 OR 15 OR 16 |
| 18 | Depression/ |
| 19 | Anxiety/ |
| 20 | Mental health/ |
| 21 | (anxiet* or anxiou* or depressi* or mental health or well being or wellbeing or mood or stress).ti,ab,kf. |
| 22 | 18 OR 19 OR 20 OR 21 |
| 23 | (program* or intervention* or trial).ti,ab,kf. |
| 24 | 10 AND 17 AND 22 AND 23 |

Note: search was applied to other Ovid databases (Embase, Emcare, PsycINFO) and adapted heading terms

### SCOPUS

(TITLE-ABS-KEY("healthy eating" or diet or "weight loss" or "weight management" or "weight reduction program" or "physical activit*" or exercis* or walk* or fitness or "sedentar* behavior" or "sitting time" or lifestyle or sleep)) AND (TITLE-ABS-KEY("cell phone" or "internet-based intervention" or "web-based intervention" or "online intervention" or mobile* or eHealth or "mobile health" or mHealth or "digital health" or online or email or website or "digital intervention" or "mobile app*" or smartphone or "mobile phone" or acceleromet* or pedomet* or wearable* or "activity monitor*" or remote or "fitness tracker*" or fitbit* or "social media" or "social network" or "text messag*" or chatbot or "artificial intelligence" or "virtual assistant")) AND (TITLE-ABS-KEY(anxiet* or anxiou* or depressi* or "mental health" or "well being" or wellbeing or mood or stress)) AND (TITLE-ABS-KEY(program* or intervention* or trial)) AND NOT (child* or adolescen* or protocol or "systematic review" or "meta?analysis" or observational)

### CINAHL

| 1 | MH("internet-based intervention" or "cellular phone" or "mobile applications" or "text messaging" or "social media" or "fitness trackers" or "wearable sensors") |
| --- | --- |
| 2 | (TI("cell phone" or "internet-based intervention" or "web-based intervention" or "online intervention" or mobile* or eHealth or "mobile health" or mHealth or "digital health" or online or email or website or "digital intervention" or "mobile app*" or smartphone or "mobile phone" or acceleromet* or pedomet* or wearable* or "activity monitor*" or remote or "fitness tracker*" or fitbit* or "social media" or "social network" or "text messag*" or chatbot or "artificial intelligence" or "virtual assistant") or (AB("cell phone" or "internet-based intervention" or "web-based intervention" or "online intervention" or mobile* or eHealth or "mobile health" or mHealth or "digital health" or online or email or website or "digital intervention" or "mobile app*" or smartphone or "mobile phone" or acceleromet* or pedomet* or wearable* or "activity monitor*" or remote or "fitness tracker*" or fitbit* or "social media" or "social network" or "text messag*" or chatbot or "artificial intelligence" or "virtual assistant")) |
| 3 | 1 OR 2 |
| 4 | MH("health behavior" or "exercise" or "physical fitness" or lifestyle, sedentary" or "sleep" or "diet") |
| 5 | (TI("healthy eating" or diet or "weight loss" or "weight management" or "weight reduction program" or "physical activit*" or exercis* or walk* or fitness or "sedentar* behavior" or "sitting time" or lifestyle or sleep) OR (AB("healthy eating" or diet or "weight loss" or "weight management" or "weight reduction program" or "physical activit*" or exercis* or walk* or fitness or "sedentar* behavior" or "sitting time" or lifestyle or sleep)) |
| 6 | 4 OR 5 |
| 7 | MH("depression" or "anxiety" or "mental health") |
| 8 | (TI(anxiet* or anxiou* or depressi* or "mental health" or "well being" or wellbeing or mood or stress) or (AB(anxiet* or anxiou* or depressi* or "mental health" or "well being" or wellbeing or mood or stress)) |
| 9 | 7 OR 8 |
| 10 | (TI("program" or "trial" or "intervention") or (AB(program* or intervention* or trial)) |
| 11 | 3 AND 6 AND 9 AND 10 |
