# Supplementary material for: Effectiveness of Digital Lifestyle Interventions on Depression, Anxiety, Stress, and Well-Being: Systematic Review and Meta-Analysis
Source: J Med Internet Res. 2025 Mar 20;27:e56975. doi: 10.2196/56975 (PMC11969127; doi:10.2196/56975)
Supplement: Multimedia Appendix 3 [file jmir_v27i1e56975_app3.docx]

# Appendix 2.

## Table 1. Reasons for exclusion at full-text screening

| **Author, year** | **Reason for exclusion** |
| --- | --- |
| Aldemir [1] 2021 | Wrong intervention |
| Altazan [2] 2019 | Wrong intervention |
| Anderson [3] 2015 | Dissertation |
| Antypas [4] 2014 | Wrong control condition |
| Ashton [5] 2017 | Wrong intervention |
| Azar [6] 2015 | Wrong intervention |
| Baez [7] 2017 | Wrong intervention |
| Baker [8] 2008 | Wrong intervention (involves counselling) |
| Baldwin [9] 2020 | Wrong intervention |
| Bannik [10] 2014 | Wrong outcome measure |
| Bantum [11] 2014 | Wrong intervention |
| Bayerle [12] 2022 | Wrong intervention |
| Beauchamp [13] 2021 | Wrong intervention |
| Bellon [14] 2015 | Wrong intervention |
| Bennell [15] 2018 | Wrong intervention |
| Bennell [16] 2022 | Wrong control condition |
| Bennett [17] 2011 | Wrong intervention |
| Benzo [18] 2022 | Conference abstract |
| Berman [19] 2009 | Wrong intervention |
| Bishay [20] 2018 | Conference abstract |
| Bisson [21] 2021 | Wrong outcome measure |
| Block [22] 2008 | Wrong outcome measure |
| Bonato [23] 2019 | Wrong outcome measure |
| Bravo-Escobar [24] 2021 | Wrong intervention |
| Brindal [25] 2016 | Conference abstract |
| Brindal [26] 2019 | Wrong outcome measure |
| Buro [27] 2022 | Dissertation |
| Cabrera-Suarez [28] 2022 | Wrong intervention |
| Cai [29] 2019 | Conference abstract |
| Callisaya [30] 2021 | Wrong outcome measure |
| Campana [31] 2021 | Conference abstract |
| Caso [32] 2021 | Wrong outcome measure |
| Chan [33] 2022 | Wrong study design |
| Cobb [34] 2014 | Wrong outcome measure |
| Coelho [35] 2018 | Wrong intervention |
| Compton [36] 2021 | Wrong control condition |
| Connolly [37] 2020 | Wrong intervention |
| Cook [38] 2007 | Wrong control condition |
| Corepal [39] 2019 | Wrong intervention |
| Curran [40] 2022 | Wrong intervention |
| DaCosta [41] 2013 | Conference abstract |
| Dale [42] 2015 | Wrong intervention |
| deNiet [43] 2012 | Wrong intervention |
| Deitz [44] 2014 | Wrong outcome measure |
| Demmin [45] 2022 | Wrong intervention |
| Doorley [46] 2022 | Wrong intervention |
| Drerup [47] 2014 | Conference abstract |
| Drew [48] 2022 | Re-analysis of Young 2021 (included) |
| Drew [49] 2022 | Wrong outcome measure |
| Duan [50] 2022 | Wrong intervention |
| Duncan [51] 2021 | Wrong study design |
| EvangelistadeLima [52] 2021 | Wrong intervention |
| Faux [53] 2018 | Conference abstract |
| Friedrich [54] 2018 | Wrong intervention |
| GarthMcKay [55] 2002 | Wrong intervention |
| George [56] 2021 | Wrong control condition |
| Gilbody [57] 2021 | Wrong intervention |
| Gokal [58] 2016 | Wrong intervention |
| Gosling [59] 2018 | Mediation analysis of Christensen 2016 (included) |
| Greene [60] 2012 | Wrong outcome measure |
| Gur [61] 2020 | Wrong outcome measure |
| Haas [62] 2017 | Wrong outcome measure |
| Haller [63] 2018 | Wrong intervention |
| Hershner [64] 2016 | Wrong outcome measure |
| Hospes [65] 2009 | Wrong intervention |
| Huberty [66] 2020 | Wrong control condition |
| Hunt [67] 2021 | Wrong intervention |
| Jane [68] 2018 | Wrong control condition |
| Johansson [69] 2021 | Wrong intervention |
| JonesBell [70] 2019 | Protocol paper |
| Jukic [71] 2020 | Wrong study design |
| Kelechi [72] 2020 | Wrong intervention |
| Kim [73] 2015 | Wrong study design |
| Kim [74] 2022 | Wrong intervention |
| King [75] 2020 | Wrong study design |
| King [76] 2020 | Wrong study design |
| Kolakowsky-Haynew [77] 2011 | Conference abstract |
| Krause [78] 2021 | Conference abstract |
| Lancee [79] 2016 | Wrong study design |
| Lao [80] 2020 | Wrong study design |
| Lee [81] 2021 | Wrong control condition |
| Lejuez [82] 2019 | Wrong intervention |
| Lennefer [83] 2020 | Wrong outcome measure |
| Levin [84] 2021 | Wrong intervention |
| Li [85] 2018 | Wrong study design |
| Li [86] 2010 | Wrong intervention |
| Li [87] 2020 | Wrong intervention |
| Li [88] 2021 | Secondary analysis/wrong intervention |
| Lokman [89] 2017 | Wrong intervention |
| Lu [90] 2022 | Wrong study design |
| Luik [91] 2020 | Follow up report of Espie 2018 (included) |
| Mailey [92] 2010 | Wrong intervention |
| Mancuso [93] 2012 | Wrong control condition |
| Martin [94] 2021 | Dissertation |
| Mascarenhas [95] 2018 | Wrong intervention |
| Mayo [96] 2014 | Wrong intervention |
| McClure [97] 2011 | Wrong intervention |
| McMurdo [98] 2010 | Wrong intervention |
| Meyer [99] 2020 | Wrong control condition |
| Moller [100] 2013 | Wrong intervention |
| Motl [101] 2017 | Wrong intervention |
| Mueller [102] 2021 | Conference abstract |
| Mueller [103] 2022 | Wrong intervention |
| Mundle [104] 2016 | Conference abstract |
| Muniswamy [105] 2022 | Wrong intervention |
| Muniswamy [106] 2021 | Wrong intervention |
| Myers [107] 2021 | Wrong outcome measure |
| Naparstek [108] 2017 | Wrong intervention |
| Nicolucci [109] 2022 | Wrong intervention |
| Overas [110] 2022 | Wrong intervention |
| Palermo [111] 2016 | Wrong outcome measure |
| Paul [112] 2013 | Wrong intervention |
| Paul [113] 2014 | Wrong intervention |
| Petersen [114] 2013 | Wrong intervention |
| Pilutti [115] 2014 | Wrong intervention (involves behavioural coaching) |
| Poettgen [116] 2015 | Wrong intervention |
| Pogosova [117] 2019 | Can’t find full text |
| Pollard [118] 2011 | Conference abstract |
| Pope [119] 2018 | Wrong outcome measure |
| Proeschold-Bell [120] 2017 | Wrong intervention |
| Quinn [121] 2011 | Wrong intervention |
| Rastogi [122] 2020 | Wrong intervention |
| Rebar [123] 2016 | Wrong control condition |
| Renfrew [124] 2020 | Wrong control condition |
| Rica [125] 2020 | Wrong intervention |
| Rodrigues [126] 2018 | Wrong intervention |
| Rollo [127] 2021 | Wrong intervention |
| Roth [128] 2022 | Conference abstract |
| Salsman [129] 2020 | Conference abstract |
| Sears [130] 2021 | Conference abstract |
| Seib [131] 2022 | Wrong intervention |
| Shariful Islam [132] 2019 | Wrong outcome measure (does not measure at baseline) |
| Sharp [133] 2016 | Wrong outcome measure |
| Sherwood [134] 2008 | Wrong study design |
| Siengsukon [135] 2021 | Wrong control condition |
| Silarova [136] 2019 | Wrong outcome measure |
| Slater [137] 2020 | Wrong study design |
| Sparrow [138] 2011 | Wrong intervention |
| Stahl [139] 2020 | Wrong intervention |
| Stapleton [140] 2020 | Wrong intervention |
| Steinberg [141] 2014 | Wrong intervention |
| Stewart [142] 2015 | Conference abstract |
| Stuckey [143] 2013 | Conference abstract |
| Su [144] 2021 | Wrong intervention |
| Sunnhed [145] 2020 | Wrong intervention |
| Tandon [146] 2022 | Wrong intervention |
| Timurtas [147] 2020 | Conference abstract |
| Van der Zweerde [148] 2019 | Wrong intervention |
| Van Straten [149] 2008 | Wrong intervention |
| Van Uytsel [150] 2022 | Wrong intervention |
| Varnfield [151] 2012 | Conference abstract |
| Vidmar [152] 2022 | Wrong intervention |
| Walsh [153] 2021 | Wrong intervention |
| Wang [154] 2015 | Dissertation |
| Watt [155] 2022 | Conference abstract |
| Weber [156] 2019 | Wrong intervention |
| Whittemore [157] 2019 | Wrong intervention |
| Wiegand [158] 2010 | Wrong intervention |
| Wilczynska [159] 2022 | Wrong intervention |
| Willems [160] 2017 | Wrong intervention (doesn’t target lifestyle behaviour) |
| Williams [161] 2010 | Wrong intervention |
| Wilson [162] 2018 | Wrong intervention |
| Yanez [163] 2020 | Wrong control condition |
| Zamorano [164] 2021 | Conference abstract |
| Zhang [165] 2021 | Wrong intervention |
| Zhao [166] 2021 | Wrong intervention |
| Zhou [167] 2020 | Wrong intervention |
| Ziolkowski [168] 2020 | Wrong intervention |

1. Aldemir, K. and A. Gürkan, *The effect of pedometer‐supported walking and telemonitoring after disc hernia surgery on pain and disability levels and quality of life.* International Journal of Nursing Practice (John Wiley & Sons, Inc.), 2021. **27**(2): p. 1-12.

2. Altazan, A.D., et al., *Mood and quality of life changes in pregnancy and postpartum and the effect of a behavioral intervention targeting excess gestational weight gain in women with overweight and obesity: a parallel-arm randomized controlled pilot trial.* BMC Pregnancy & Childbirth, 2019. **19**(1): p. N.PAG-N.PAG.

3. Anderson, D.R., et al., *Lower anxiety associated with greater physical activity in a pedometer-based intervention among cardiac patients.* Psychosomatic Medicine, 2015. **77**(3): p. A17-A18.

4. Antypas, K. and S.C. Wangberg, *An Internet- and mobile-based tailored intervention to enhance maintenance of physical activity after cardiac rehabilitation: short-term results of a randomized controlled trial.* Journal of medical Internet research, 2014. **16**(3): p. e77.

5. Ashton, L.M., et al., *Feasibility and preliminary efficacy of the 'HEYMAN' healthy lifestyle program for young men: a pilot randomised controlled trial.* Nutrition Journal, 2017. **16**: p. 1-17.

6. Azar, K.M., et al., *Electronic cardiometabolic program (ECMP): A randomized controlled trial.* Diabetes, 2015. **64**(SUPPL. 1): p. A226.

7. Baez, M., et al., *Effects of online group exercises for older adults on physical, psychological and social wellbeing: a randomized pilot trial.* PeerJ, 2017. **5**(101603425): p. e3150.

8. Baker, G., et al., *The effect of a pedometer-based community walking intervention "Walking for Wellbeing in the West" on physical activity levels and health outcomes: A 12-week randomized controlled trial.* The International Journal of Behavioral Nutrition and Physical Activity, 2008. **5**(Ainsworth, B. E., Haskell, W. L., Whitt, M. C., Irwin, M. L., Swartz, A. M., Strath, S. J., O'Brien, W. L., Bassett, D. R., Jr.., Schmitz, K. H., Emplaincourt, P. O., et al. (2000). Compendium of physical activities: an update of activity codes and MET in).

9. Baldwin, P.A., et al., *A Web-Based Mental Health Intervention to Improve Social and Occupational Functioning in Adults With Type 2 Diabetes (The Springboard Trial): 12-Month Outcomes of a Randomized Controlled Trial.* Journal of medical Internet research, 2020. **22**(12): p. e16729.

10. Bannink, R., et al., *Effectiveness of a Web-based tailored intervention (E-health4Uth) and consultation to promote adolescents' health: randomized controlled trial.* Journal of medical Internet research, 2014. **16**(5): p. e143.

11. Bantum, E.O.C., et al., *Surviving and thriving with cancer using a Web-based health behavior change intervention: randomized controlled trial.* Journal of Medical Internet Research, 2014. **16**(2): p. e54-12.

12. Bayerle, P., et al., *Effectiveness of wearable devices as a support strategy for maintaining physical activity after a structured exercise intervention for employees with metabolic syndrome: a randomized controlled trial.* BMC Sports Science, Medicine and Rehabilitation, 2022. **14**(1): p. 24.

13. Beauchamp, M.R., et al., *Online-Delivered Group and Personal Exercise Programs to Support Low Active Older Adults' Mental Health During the COVID-19 Pandemic: A Randomized Controlled Trial.* Journal of Medical Internet Research, 2021. **23**(7): p. N.PAG-N.PAG.

14. Bellon, K., et al., *A home-based walking study to ameliorate perceived stress and depressive symptoms in people with a traumatic brain injury.* Brain Injury, 2015. **29**(3): p. 313-319.

15. Bennell, K.L., et al., *Effects of internet-based pain coping skills training before home exercise for individuals with hip osteoarthritis (HOPE trial): a randomised controlled trial.* PAIN, 2018. **159**(9): p. 1833-1842.

16. Bennell, K.L., et al., *Effectiveness of an Unsupervised Online Yoga Program on Pain and Function in People With Knee Osteoarthritis : A Randomized Clinical Trial.* Annals of Internal Medicine, 2022. **175**(10): p. 1345-1355.

17. Bennett, J.B., et al., *A web-based approach to address cardiovascular risks in managers: results of a randomized trial.* Journal of occupational and environmental medicine, 2011. **53**(8): p. 911-8.

18. Benzo, R., et al., *Effect of Home-Based Rehabilitation with Health Coaching on Chronic Obstructive Pulmonary Disease Outcomes: A Randomized Study.* American Journal of Respiratory and Critical Care Medicine, 2022. **205**(1).

19. Berman, R.L.H., et al., *The effectiveness of an online mind-body intervention for older adults with chronic pain.* The journal of pain, 2009. **10**(1): p. 68-79.

20. Bishay, L.C., et al., *Effect of a wearable fitness tracker on exercise tolerance for adults with cystic fibrosis: A pilot randomized clinical trial.* Pediatric Pulmonology, 2018. **53**(Supplement 2): p. 345.

21. Bisson, A.N., V. Sorrentino, and M.E. Lachman, *Walking and Daily Affect Among Sedentary Older Adults Measured Using the StepMATE App: Pilot Randomized Controlled Trial.* JMIR mHealth and uHealth, 2021. **9**(12): p. e27208.

22. Block, G., et al., *Development of Alive! (A Lifestyle Intervention Via Email), and its effect on health-related quality of life, presenteeism, and other behavioral outcomes: randomized controlled trial.* Journal of medical Internet research, 2008. **10**(4): p. e43.

23. Bonato, M., et al., *A Mobile Application for Exercise Intervention in People Living with HIV.* Medicine and Science in Sports and Exercise, 2020. **52**(2): p. 425-433.

24. Bravo-Escobar, R., et al., *Effectiveness of e-Health cardiac rehabilitation program on quality of life associated with symptoms of anxiety and depression in moderate-risk patients.* Scientific reports, 2021. **11**(1): p. 3760.

25. Brindal, E., et al., *Results of a randomised control trial evaluating a 24-week weight maintenance intervention delivered Via a Smartphone application.* Obesity Reviews, 2016. **17**(SUPPL. 2): p. 165.

26. Brindal, E., et al., *A Mobile Phone App Designed to Support Weight Loss Maintenance and Well-Being (MotiMate): Randomized Controlled Trial.* Journal of Medical Internet Research, 2019. **21**(9): p. N.PAG-N.PAG.

27. Buro, A.W., *Feasibility of a virtual group nutrition intervention for adolescents with autism spectrum disorder.* Dissertation Abstracts International: Section B: The Sciences and Engineering, 2022. **83**(1-B): p. No-Specified.

28. Cabrera-Suarez, B., et al., *'Effectiveness of a remote nutritional intervention to increase the adherence to the Mediterranean diet among recovered depression patients'.* Nutritional Neuroscience, 2022((Cabrera-Suarez, Hernandez, Florido-Rodriguez) Psychiatry and Clinical Psychology Service, Hospital Universitario de Gran Canaria Dr. Negrin, Las Palmas de Gran Canaria, Spain(Cabrera-Suarez, Hernandez) Universidad de Las Palmas de Gran Canaria (ULPGC), L).

29. Cai, C., et al., *Home-based cardiac rehabilitation versus conventional care for patients with atrial fibrillation treated with catheter ablation: A randomized controlled trial.* Journal of Arrhythmia, 2019. **35**(Supplement 1): p. 95-96.

30. Callisaya, M.L., et al., *A novel cognitive-motor exercise program delivered via a tablet to improve mobility in older people with cognitive impairment - StandingTall Cognition and Mobility.* Experimental Gerontology, 2021. **152**((Callisaya, Vaidya, Srikanth) Peninsula Clinical School, Central Clinical School, Monash University, 2 Hastings Road, Frankston, Victoria, Australia(Callisaya, Jayakody) Menzies Institute for Medical Research, University of Tasmania, 17 Liverpool Street): p. 111434.

31. Campana, R., et al., *Efficacy and safety of an early individualized, low costs physical activity program for elderly patients affected by myocardial infarction.* European Heart Journal, Supplement, 2021. **23**(SUPPL C): p. C109-C110.

32. Caso, D., et al., *Using Messages Targeting Psychological versus Physical Health Benefits to Promote Walking Behaviour: A Randomised Controlled Trial.* Applied psychology. Health and well-being, 2021. **13**(1): p. 152-173.

33. Chan, N.Y., et al., *Efficacy of Email-delivered Versus Face-to-face Group Cognitive Behavioral Therapy for Insomnia in Youths: A Randomized Controlled Trial.* Journal of Adolescent Health, 2022. **70**(5): p. 763-773.

34. Cobb, N.K. and J. Poirier, *Effectiveness of a multimodal online well-being intervention: a randomized controlled trial.* American Journal of Preventive Medicine, 2014. **46**(1): p. 41-48.

35. Coelho, C.M., et al., *Effects of an unsupervised pedometer-based physical activity program on daily steps of adults with moderate to severe asthma: a randomized controlled trial.* Journal of Sports Sciences, 2018. **36**(10): p. 1186-1193.

36. Compton, P., et al., *A randomized controlled trial to evaluate a behavioral economic strategy for improving mobility in veterans with chronic pain.* PLoS ONE, 2021. **16**(10).

37. Connolly, L.J., et al., *Impact of a novel home-based exercise intervention on health indicators in inactive premenopausal women: a 12-week randomised controlled trial.* European journal of applied physiology, 2020. **120**(4): p. 771-782.

38. Cook, R.F., et al., *A field test of web-based workplace health promotion program to improve dietary practices, reduce stress, and increase physical activity: Randomized controlled trial.* Journal of Medical Internet Research, 2007. **9**(2): p. 1-14.

39. Corepal, R., et al., *A feasibility study of 'The StepSmart Challenge' to promote physical activity in adolescents.* Pilot and feasibility studies, 2019. **5**(101676536): p. 132.

40. Curran, M., et al., *Steps Ahead: Optimising physical activity in adults with cystic fibrosis: A pilot randomised trial using wearable technology, goal setting and text message feedback.* Journal of Cystic Fibrosis, 2022((Curran, Tierney, Jurascheck, Cahalan) School of Allied Health, University of Limerick, Limerick, Ireland(Curran, Collins, Kennedy, McDonnell, Casserly) University Hospital Limerick, Limerick, Ireland(Curran, Tierney, Sheikhi, Walsh, Cahalan) Health Resea).

41. Da Costa, D., I. Lowensteyn, and S. Khalife, *Efficacy of a home-based motivationallytailored exercise intervention for increasing physical activity during pregnancy.* Psychosomatic Medicine, 2013. **75**(3): p. A104-A105.

42. Dale, L.P., et al., *Text Message and Internet Support for Coronary Heart Disease Self-Management: Results From the Text4Heart Randomized Controlled Trial.* Journal of Medical Internet Research, 2015. **17**(10): p. e237-11.

43. de Niet, J., et al., *The effect of a short message service maintenance treatment on body mass index and psychological well-being in overweight and obese children: A randomized controlled trial.* Pediatric Obesity, 2012. **7**(3): p. 205-219.

44. Deitz, D., et al., *Heart Healthy Online: An Innovative Approach to Risk Reduction in the Workplace.* Journal of Occupational & Environmental Medicine, 2014. **56**(5): p. 547-553.

45. Demmin, D.L., S.M. Silverstein, and T.J. Shors, *Mental and physical training with meditation and aerobic exercise improved mental health and well-being in teachers during the COVID-19 pandemic.* Frontiers in Human Neuroscience, 2022. **16**(Abos, A, Sevil-Serrano, J., Julian-Clemente, J. A., Generelo, E., & Garcia-Gonzalez, L. (2021). Improving teachers' work-related outcomes through a group-based physical activity intervention during leisure-time. J. Exp. Educ., 89, 306-325. <http://dx.doi.o>).

46. Doorley, J.D., et al., *Feasibility Randomized Controlled Trial of a Mind-Body Activity Program for Older Adults With Chronic Pain and Cognitive Decline: The Virtual "Active Brains" Study.* The Gerontologist, 2022. **62**(7): p. 1082-1094.

47. Drerup, M.L., et al., *Impact of the web-based cognitive behavioral therapy program on insomnia symptoms and perceived stress: Results of a randomized controlled trial.* Sleep, 2014. **37**(SUPPL. 1): p. A173.

48. Drew, R.J., P.J. Morgan, and M.D. Young, *Mechanisms of an eHealth program targeting depression in men with overweight or obesity: A randomised trial.* Journal of affective disorders, 2022. **299**(h3v, 7906073): p. 309-317.

49. Drew, R.J., et al., *Behavioral and Cognitive Outcomes of an Online Weight Loss Program for Men With Low Mood: A Randomized Controlled Trial.* Annals of Behavioral Medicine, 2022. **56**(10): p. 1026-1041.

50. Duan, Y., et al., *The Effectiveness of Sequentially Delivered Web-Based Interventions on Promoting Physical Activity and Fruit-Vegetable Consumption among Chinese College Students: Mixed Methods Study.* Journal of Medical Internet Research, 2022. **24**(1): p. e30566.

51. Duncan, M.J., et al., *Effect of a physical activity and sleep m-health intervention on a composite activity-sleep behaviour score and mental health: a mediation analysis of two randomised controlled trials.* The international journal of behavioral nutrition and physical activity, 2021. **18**(1): p. 45.

52. Evangelista de Lima, B., et al., *Effects of Xbox Kinect exercise training on sleep quality, anxiety and functional capacity in older adults.* Journal of bodywork and movement therapies, 2021. **28**(9700068): p. 271-275.

53. Faux, S.G., et al., *'Reboot Online': A randomised controlled trial demonstrating that an internet-delivered multidsipclinary pain management program is effective in chronic pain.* Neurorehabilitation and Neural Repair, 2018. **32**(4-5): p. 354-355.

54. Friedrich, A., M. Classen, and A.A. Schlarb, *Sleep better, feel better? Effects of a CBT-I and HT-I sleep training on mental health, quality of life and stress coping in university students: A randomized pilot controlled trial.* BMC Psychiatry, 2018. **18**(1): p. 268.

55. Garth McKay, H., et al., *Internet-based diabetes self-management and support: Initial outcomes from the diabetes network project.* Rehabilitation Psychology, 2002. **47**(1): p. 31-48.

56. George, N., et al., *Functional impairment among individuals with mood disorders at risk for cardiovascular disease.* Bipolar Disorders, 2021. **23**(SUPPL 1): p. 88.

57. Gilbody, S., et al., *Behavioural activation to prevent depression and loneliness among socially isolated older people with long-term conditions: The BASIL COVID-19 pilot randomised controlled trial.* PLoS medicine, 2021. **18**(10): p. e1003779.

58. Gokal, K., et al., *Effects of a self-managed home-based walking intervention on psychosocial health outcomes for breast cancer patients receiving chemotherapy: a randomised controlled trial.* Supportive Care in Cancer, 2016. **24**(3): p. 1139-1166.

59. Gosling, J.A., et al., *Online insomnia treatment and the reduction of anxiety symptoms as a secondary outcome in a randomised controlled trial: The role of cognitive-behavioural factors.* Australian & New Zealand Journal of Psychiatry, 2018. **52**(12): p. 1183-1193.

60. Greene, G.W., et al., *Impact of an online healthful eating and physical activity program for college students.* American Journal of Health Promotion, 2012. **27**(2): p. e47-58.

61. Gur, F., G.C. Gur, and V. Ayan, *The Effect of the ERVE Smartphone App on Physical Activity, Quality of Life, Self-Efficacy, and Exercise Motivation for Inactive People: A Randomized Controlled Trial.* European Journal of Integrative Medicine, 2020. **39**((Gur) University of Pamukkale, Faculty of Sport Science, Department of Coaching Education, Denizli 20000, Turkey(Gur) University of Pamukkale, Faculty of Health Science, Department of Nursing, Denizli 20000, Turkey(Ayan) University of Trabzon, Faculty of): p. 101198.

62. Haas, K., A. Martin, and K.T. Park, *Text Message Intervention (TEACH) Improves Quality of Life and Patient Activation in Celiac Disease: A Randomized Clinical Trial.* Journal of Pediatrics, 2017. **185**: p. 62-67.e2.

63. Haller, N., et al., *Individualized web-Based exercise for the treatment of depression: Randomized controlled trial.* JMIR Mental Health, 2018. **5**(10): p. e10698.

64. Hershner, S.D. and L. O'Brien, *Sleep to stay awake: An online sleep education intervention.* Sleep, 2016. **39**(SUPPL. 1): p. A382.

65. Hospes, G., et al., *Enhancement of daily physical activity increases physical fitness of outclinic COPD patients: Results of an exercise counseling program.* Patient Education & Counseling, 2009. **75**(2): p. 274-278.

66. Huberty, J., et al., *Online yoga to reduce post traumatic stress in women who have experienced stillbirth: a randomized control feasibility trial.* BMC Complementary Medicine & Therapies, 2020. **20**(1): p. 1-19.

67. Hunt, M., et al., *Efficacy of Zemedy, a Mobile Digital Therapeutic for the Self-management of Irritable Bowel Syndrome: Crossover Randomized Controlled Trial.* JMIR mHealth and uHealth, 2021. **9**(5): p. e26152.

68. Jane, M., et al., *Psychological effects of belonging to a Facebook weight management group in overweight and obese adults: Results of a randomised controlled trial.* Health & Social Care in the Community, 2018. **26**(5): p. 714-724.

69. Johansson, P., et al., *The impact of internet-based cognitive behavioral therapy and depressive symptoms on self-care behavior in patients with heart failure: A secondary analysis of a randomised controlled trial.* International Journal of Nursing Studies, 2021. **116**: p. N.PAG-N.PAG.

70. Jones Bell, M., et al., *Healthy Teens @ School: Evaluating and disseminating transdiagnostic preventive interventions for eating disorders and obesity for adolescents in school settings.* Internet Interventions, 2019. **16**((Jones Bell) Stanford University School of Medicine, Department of Psychiatry and Behavioral Sciences, 401 Quarry Road, Stanford, CA 94301, United States(Jones Bell, Zeiler, Karwautz, Wagner) Medical University of Vienna, Department for Child and Adolesce): p. 65-75.

71. Jukic, T., et al., *The effect of active occupational stress management on psychosocial and physiological wellbeing: a pilot study.* BMC medical informatics and decision making, 2020. **20**(1): p. 321.

72. Kelechi, T.J., et al., *FOOTFIT Physical Activity mHealth Intervention for Minimally Ambulatory Individuals With Venous Leg Ulcers: A Randomized Controlled Trial.* Journal of Wound, Ostomy & Continence Nursing, 2020. **47**(2): p. 173-181.

73. Kim, C.-J., et al., *Effects of an internet-based lifestyle intervention on cardio-metabolic risks and stress in Korean workers with metabolic syndrome: a controlled trial.* Patient Education & Counseling, 2015. **98**(1): p. 111-119.

74. Kim, H.B. and A.H. Hyun, *Psychological and Biochemical Effects of an Online Pilates Intervention in Pregnant Women during COVID-19: A Randomized Pilot Study.* International Journal of Environmental Research and Public Health, 2022. **19**(17): p. 10931.

75. King, A.C., et al., *Testing the effectiveness of physical activity advice delivered via text messaging vs. human phone advisors in a Latino population: The On The Move randomized controlled trial design and methods.* Contemporary Clinical Trials, 2020. **95**((King) Department of Epidemiology & Population Health, Stanford University School of Medicine, Stanford, CA 94305, United States(King, Campero, Sheats, Castro Sweet, Espinosa, Garcia, Hauser, Done, Patel, Ahn) Stanford Prevention Research Center, Departme): p. 106084.

76. King, A.C., et al., *Effects of Counseling by Peer Human Advisors vs Computers to Increase Walking in Underserved Populations: The COMPASS Randomized Clinical Trial.* JAMA Internal Medicine, 2020. **180**(11): p. 1481-1490.

77. Kolakowsky-Hayner, S., et al., *Stepping-out: Psychosocial and functional impact of a 12-week home-based physical activity program after brain injury.* Archives of Physical Medicine and Rehabilitation, 2011. **92**(10): p. 1692.

78. Krause, K., et al., *An individualized proactive e-health intervention promoting a lifestyle against depression:Results of a randomized controlled trial over 24 months.* Gesundheitswesen, Supplement, 2021. **83**(8-9): p. 709.

79. Lancee, J., et al., *Guided Online or Face-to-Face Cognitive Behavioral Treatment for Insomnia: A Randomized Wait-List Controlled Trial.* Sleep, 2016. **39**(1): p. 183-91.

80. Lao, S.S.W., S.Y. Chair, and M.L.T. Leong, *The effects of smartphone-based cardiac rehabilitation program for percutaneous coronary intervention patients in macau.* Journal of the Hong Kong College of Cardiology, 2020. **28**(2): p. 83.

81. Lee, D., *Preliminary efficacy of a gamified, behavior change techniques-based mobile application puzzlewalk on increasing physical activity and reducing anxiety in adults with autism spectrum disorder.* Dissertation Abstracts International: Section B: The Sciences and Engineering, 2021. **82**(2-B): p. No-Specified.

82. Lejuez, C.W., et al., *Pilot Randomized Trial of a Self-Help Behavioral Activation Mobile App for Utilization in Primary Care.* Behavior Therapy, 2019. **50**(4): p. 817-827.

83. Lennefer, T., et al., *Improving employees' work-related well-being and physical health through a technology-based physical activity intervention: A randomized intervention-control group study.* Journal of Occupational Health Psychology, 2020. **25**(2): p. 143-158.

84. Levin, M.E., et al., *A randomized controlled trial of online acceptance and commitment therapy to improve diet and physical activity among adults who are overweight/obese.* Translational Behavioral Medicine, 2021. **11**(6): p. 1216-1225.

85. Li, J., et al., *Exergames vs. traditional exercise: investigating the influencing mechanism of platform effect on subthreshold depression among older adults.* Aging & mental health, 2018. **22**(12): p. 1634-1641.

86. Li, L., et al., *Improving the health and mental health of people living with HIV/AIDS: 12-month assessment of a behavioral intervention in Thailand.* American journal of public health, 2010. **100**(12): p. 2418-25.

87. Li, Y. and M.Y.M. Tse, *An Online Pain Education Program for Working Adults: Pilot Randomized Controlled Trial.* Journal of Medical Internet Research, 2020. **22**(1): p. N.PAG-N.PAG.

88. Li, Y., et al., *Mediating Effects of Stigma and Depressive Symptoms in a Social Media-Based Intervention to Improve Long-term Quality of Life Among People Living With HIV: Secondary Analysis of a Randomized Controlled Trial.* Journal of medical Internet research, 2021. **23**(11): p. e27897.

89. Lokman, S., et al., *Complaint-Directed Mini-Interventions for Depressive Complaints: A Randomized Controlled Trial of Unguided Web-Based Self-Help Interventions.* Journal of medical Internet research, 2017. **19**(1): p. e4.

90. Lu, Y.T. and Y. Wu, *The effect of an instant message‐based lifestyle and stress management intervention on the reduction of cardiovascular disease risk.* International Journal of Nursing Practice (John Wiley & Sons, Inc.), 2022. **28**(6): p. 1-11.

91. Luik, A.I., et al., *Long-term benefits of digital cognitive behavioural therapy for insomnia: Follow-up report from a randomized clinical trial.* Journal of Sleep Research, 2020. **29**(4).

92. Mailey, E.L., et al., *Internet-delivered physical activity intervention for college students with mental health disorders: a randomized pilot trial.* Psychology, health & medicine, 2010. **15**(6): p. 646-59.

93. Mancuso, C.A., et al., *Increasing physical activity in patients with asthma through positive affect and self-affirmation: A randomized trial.* Archives of Internal Medicine, 2012. **172**(4): p. 337-343.

94. Martin, A.C., *The efficacy of online vinyasa yoga and TaijifitTM on physical health outcome measures and quality of live of adult informal caregivers.* Dissertation Abstracts International: Section B: The Sciences and Engineering, 2021. **82**(11-B): p. No-Specified.

95. Mascarenhas, M.N., et al., *Increasing Physical Activity in Mothers Using Video Exercise Groups and Exercise Mobile Apps: Randomized Controlled Trial.* Journal of Medical Internet Research, 2018. **20**(5): p. 1-1.

96. Mayo, N.E., et al., *Pedometer-facilitated walking intervention shows promising effectiveness for reducing cancer fatigue: A pilot randomized trial.* Clinical Rehabilitation, 2014. **28**(12): p. 1198-1209.

97. McClure, J.B., et al., *Feasibility and acceptability of a multiple risk factor intervention: the Step Up randomized pilot trial.* BMC public health, 2011. **11**(100968562): p. 167.

98. McMurdo, M.E.T., et al., *Do pedometers increase physical activity in sedentary older women? A randomized controlled trial.* Journal of the American Geriatrics Society, 2010. **58**(11): p. 2099-2106.

99. Meyer, J.D., et al., *Feasibility of an Exercise and CBT Intervention for Treatment of Depression: A Pilot Randomized Controlled Trial.* Frontiers in Psychiatry, 2022. **13**((Meyer, Perkins, Brower, Lansing, Slocum, Lee) Department of Kinesiology, Iowa State University, Ames, IA, United States(Slocum, Wade) Department of Psychology, Iowa State University, Ames, IA, United States(Thomas) Department of Psychological and Brain S): p. 799600.

100. Moller, T., et al., *At cancer diagnosis: A 'window of opportunity' for behavioural change towards physical activity. A randomised feasibility study in patients with colon and breast cancer.* BMJ Open, 2013. **3**(11): p. e003556.

101. Motl, R.W., et al., *Randomized controlled trial of an e-learning designed behavioral intervention for increasing physical activity behavior in multiple sclerosis.* Multiple Sclerosis Journal - Experimental, Translational and Clinical, 2017. **3**(4).

102. Mueller, J., et al., *Supporting Weight Management during COVID-19 (SWiM-C): A randomised controlled trial of an acceptance-based behavioural intervention to prevent weight gain during the pandemic.* Obesity Facts, 2021. **14**(SUPPL 1): p. 189.

103. Mueller, J., et al., *Supporting Weight Management during COVID-19 (SWiM-C): twelve-month follow-up of a randomised controlled trial of a web-based, ACT-based, guided self-help intervention.* International journal of obesity (2005), 2022(101256108).

104. Mundle, J.S., et al., *Assessing the impact of accelerometry device use on exercise motivation and clinical outcomes in patients attending cardiac rehabilitation following percutaneous coronary intervention or cardiac surgery.* Journal of Cardiopulmonary Rehabilitation and Prevention, 2016. **36**(5): p. 386.

105. Muniswamy, P., et al., *Short-term effects of a social media-based intervention on the physical and mental health of remotely working young software professionals: A randomised controlled trial.* Applied psychology. Health and well-being, 2022. **14**(2): p. 537-554.

106. Muniswamy, P., et al., *Short-term effects of a social media-based intervention on the physical and mental health of remotely working young software professionals: A randomised controlled trial.* Applied Psychology: Health and Well-Being, 2021: p. No-Specified.

107. Myers, N.D., et al., *Effectiveness of the Fun For Wellness online behavioral intervention to promote subjective well-being in adults with obesity: A randomized controlled trial.* Journal of Happiness Studies: An Interdisciplinary Forum on Subjective Well-Being, 2021. **22**(4): p. 1905-1923.

108. Naparstek, J., et al., *Internet-delivered obesity treatment improves symptoms of and risk for depression.* Obesity (Silver Spring, Md.), 2017. **25**(4): p. 671-675.

109. Nicolucci, A., et al., *Effect of a Behavioural Intervention for Adoption and Maintenance of a Physically Active Lifestyle on Psychological Well-Being and Quality of Life in Patients with Type 2 Diabetes: The IDES_2 Randomized Clinical Trial.* Sports Medicine, 2022. **52**(3): p. 643-654.

110. Overas, C.K., et al., *Multimorbidity and co-occurring musculoskeletal pain do not modify the effect of the selfBACK app on low back pain-related disability.* BMC Medicine, 2022. **20**(1): p. 53.

111. Palermo, T.M., et al., *Internet-delivered cognitive-behavioral treatment for adolescents with chronic pain and their parents: a randomized controlled multicenter trial.* PAIN, 2016. **157**(1): p. 174-185.

112. Paul, L., et al., *Physiotherapy-led web-based rehabilitation for people with multiple sclerosis.* Multiple Sclerosis, 2013. **19**(11 SUPPL. 1): p. 555-556.

113. Paul, L., et al., *Web-based physiotherapy for people moderately affected with Multiple Sclerosis; Quantitative and qualitative data from a randomized, controlled pilot study.* Clinical Rehabilitation, 2014. **28**(9): p. 924-935.

114. Petersen, C.B., M. Gronbaek, and J.S. Tolstrup, *The effect of a pedometer-based physical activity intervention on health outcomes: A 3-month randomised controlled trial.* European Journal of Epidemiology, 2013. **28**(1 SUPPL. 1): p. S220.

115. Pilutti, L.A., et al., *Randomized controlled trial of a behavioral intervention targeting symptoms and physical activity in multiple sclerosis.* Multiple sclerosis (Houndmills, Basingstoke, England), 2014. **20**(5): p. 594-601.

116. Poettgen, J., et al., *Online fatigue management program for patients with multiple sclerosis-a randomized controlled trial.* Multiple Sclerosis, 2015. **23**(11 SUPPL. 1): p. 41-42.

117. Pogosova, N.V., et al., *The Impact of Secondary Prevention Programs Incorporating Remote Technologies on Psychological Well-Being and Quality of Life in Coronary Heart Disease Patients with Abdominal Obesity.* Kardiologiia, 2019. **59**(12): p. 11-19.

118. Pollard, A., et al., *The role of psychosocial interventions in modifying health behaviours: Findings from a randomised controlled pilot to test the effects of a behavioural intervention (Theory-Based Information and Advice) on uptake of physical activity in breast cancer surv.* Psycho-Oncology, 2011. **20**(SUPPL. 2): p. 88.

119. Pope, Z.C., et al., *Effectiveness of combined smartwatch and social media intervention on breast cancer survivor health outcomes: A 10-week pilot randomized trial.* Journal of Clinical Medicine, 2018. **7**(6): p. 140.

120. Proeschold-Bell, R.J., et al., *A 2-Year Holistic Health and Stress Intervention: Results of an RCT in Clergy.* American journal of preventive medicine, 2017. **53**(3): p. 290-299.

121. Quinn, C.C., et al., *Cluster-randomized trial of a mobile phone personalized behavioral intervention for blood glucose control.* Diabetes Care, 2011. **34**(9): p. 1934-1942.

122. Rastogi, S., et al., *Effect of a technology-supported physical activity intervention on health-related quality of life, sleep, and processes of behavior change in cancer survivors: A randomized controlled trial.* Psycho-oncology, 2020. **29**(11): p. 1917-1926.

123. Rebar, A.L., et al., *Healthy mind, healthy body: A randomized trial testing the efficacy of a computer-tailored vs. interactive web-based intervention for increasing physical activity and reducing depressive symptoms.* Mental Health and Physical Activity, 2016. **11**(Ajzen, I. (1991). The theory of planned behaviour. Organizational Behavior and Human Decision Processes, 50, 179-211. <http://dx.doi.org/10.1016/0749-5978(91)90020-TAustralian> Bureau of Statistics. (2009). Mental health, 13-18. Canberra: Australian Institu): p. 29-37.

124. Renfrew, M.E., et al., *A Web- and Mobile App-Based Mental Health Promotion Intervention Comparing Email, Short Message Service, and Videoconferencing Support for a Healthy Cohort: Randomized Comparative Study.* Journal of medical Internet research, 2020. **22**(1): p. e15592.

125. Rica, R.L., et al., *Effects of a Kinect-based physical training program on body composition, functional fitness and depression in institutionalized older adults.* Geriatrics & gerontology international, 2020. **20**(3): p. 195-200.

126. Rodrigues, E.V., et al., *Effects of Dance Exergaming on Depressive Symptoms, Fear of Falling, and Musculoskeletal Function in Fallers and Nonfallers Community-Dwelling Older Women.* Rejuvenation research, 2018. **21**(6): p. 518-526.

127. Rollo, S. and H. Prapavessis, *A combined health action process approach and mHealth intervention to reduce workplace sitting time in office-working adults: a secondary analysis examining health-related quality of life and work performance outcomes.* Psychology & Health, 2021. **36**(10): p. 1200-1216.

128. Roth, L. and A. Horstmann, *The evaluation of zanadio - a digital health application for people with obesity.* Obesity Facts, 2022. **15**(Supplement 1): p. 282.

129. Salsman, J., et al., *A randomized pilot trial of an mhealth physical activity intervention for adolescent and young adult cancer survivors: Feasibility, acceptability, and psychological well-being outcomes.* Psycho-Oncology, 2020. **29**(Supplement 1): p. 90-91.

130. Sears, L.E., et al., *One drop digital app and coaching improves lifestyle risks, glycemic control and psychological wellbeing in people with hypertension and type 2 diabetes.* Circulation, 2021. **144**(SUPPL 1).

131. Seib, C., et al., *Improving health-related quality of life in women with breast, blood, and gynaecological Cancer with an eHealth-enabled 12-week lifestyle intervention: the women's wellness after Cancer program randomised controlled trial.* BMC cancer, 2022. **22**(1): p. 747.

132. Shariful Islam, S.M., et al., *Effect of text messaging on depression in patients with coronary heart disease: a substudy analysis from the TEXT ME randomised controlled trial.* BMJ open, 2019. **9**(2): p. e022637.

133. Sharp, P. and C. Caperchione, *The effects of a pedometer-based intervention on first-year university students: A randomized control trial.* Journal of American College Health, 2016. **64**(8): p. 630-638.

134. Sherwood, N.E., et al., *A new approach to physical activity maintenance: rationale, design, and baseline data from the Keep Active Minnesota Trial.* BMC Geriatrics, 2008. **8**: p. 17-17.

135. Siengsukon, C.F., E. Silveira Beck Jr, and M. Drerup, *Feasibility and Treatment Effect of a Web-Based Cognitive Behavioral Therapy for Insomnia Program in Individuals with Multiple Sclerosis: A Pilot Randomized Controlled Trial.* International Journal of MS Care, 2021. **23**(3): p. 107-113.

136. Silarova, B., et al., *Effect of communicating phenotypic and genetic risk of coronary heart disease alongside web-based lifestyle advice: The INFORM Randomised Controlled Trial.* Heart, 2019. **105**(13): p. 982-989.

137. Slater, H., et al., *Evaluation of Digital Technologies Tailored to Support Young People's Self-Management of Musculoskeletal Pain: Mixed Methods Study.* Journal of Medical Internet Research, 2020. **22**(6): p. N.PAG-N.PAG.

138. Sparrow, D., et al., *Increases in muscle strength and balance using a resistance training program administered via a telecommunications system in older adults.* The journals of gerontology. Series A, Biological sciences and medical sciences, 2011. **66**(11): p. 1251-7.

139. Stahl, S.T., et al., *Digital Monitoring of Sleep, Meals, and Physical Activity for Reducing Depression in Older Spousally-Bereaved Adults: A Pilot Randomized Controlled Trial.* American Journal of Geriatric Psychiatry, 2020. **28**(10): p. 1102-1106.

140. Stapleton, P., et al., *Online Delivery of Emotional Freedom Techniques for Food Cravings and Weight Management: 2-Year Follow-Up.* Journal of Alternative & Complementary Medicine, 2020. **26**(2): p. 98-106.

141. Steinberg, D.M., et al., *The effect of a "maintain, don't gain" approach to weight management on depression among black women: results from a randomized controlled trial.* American journal of public health, 2014. **104**(9): p. 1766-73.

142. Stewart, K.J., et al., *A mobile health driven walking program improves quality of life but not fitness or fatness in obese sedentary women.* Circulation, 2015. **132**(SUPPL. 3).

143. Stuckey, M.I., et al., *Does prescriptive exercise with mobile health tracking improve self-efficacy and health status in individuals with metabolic syndrome?* Circulation, 2013. **128**(22 SUPPL. 1).

144. Su, J.J. and D.S.F. Yu, *Effects of a nurse-led eHealth cardiac rehabilitation programme on health outcomes of patients with coronary heart disease: A randomised controlled trial.* International Journal of Nursing Studies, 2021. **122**((Su) WHO Collaborating Centre for Community Health Services (WHOCC), School of Nursing, The Hong Kong Polytechnic University, Hong Kong Special Administrative Region, China(Yu) School of Nursing, Li Ka Shing Faculty of Medicine, The University of Hong Kon): p. 104040.

145. Sunnhed, R., et al., *Comparing internet-delivered cognitive therapy and behavior therapy with telephone support for insomnia disorder: A randomized controlled trial.* Sleep, 2020. **43**(2).

146. Tandon, P., et al., *Heal-me PiONEer (personalized online nutrition and exercise): An RCT assessing 2 levels of app-based programming in individuals with chronic disease.* Contemporary Clinical Trials, 2022. **118**((Tandon, Ismond, Cruz, Hyde, Bhanji) Department of Medicine, Division of Gastroenterology, University of Alberta, Edmonton, AB, Canada(Purdy, Suderman, McNeely) Department of Physical Therapy, University of Alberta, Edmonton, AB, Canada(Etruw, Stickland): p. 106791.

147. Timurtas, E., et al., *Effects of supported exercise training on health-related quality of life in patients with type 2 diabetes.* Quality of Life Research, 2020. **29**(SUPPL 1): p. S181.

148. van der Zweerde, T., et al., *Does online insomnia treatment reduce depressive symptoms? A randomized controlled trial in individuals with both insomnia and depressive symptoms.* Psychological Medicine, 2019. **49**(3): p. 501-509.

149. van Straten, A., P. Cuijpers, and N. Smits, *Effectiveness of a web-based self-help intervention for symptoms of depression, anxiety, and stress: randomized controlled trial.* Journal of medical Internet research, 2008. **10**(1): p. e7.

150. Van Uytsel, H., et al., *Effect of the e-health supported INTER-ACT lifestyle intervention on postpartum weight retention and body composition, and associations with lifestyle behavior: A randomized controlled trial.* Preventive medicine, 2022. **164**(pm4, 0322116): p. 107321.

151. Varnfield, M., et al., *Technology based home-care model improves outcomes of uptake, adherence and health in cardiac rehabilitation.* European Heart Journal, 2012. **33**(SUPPL. 1): p. 445.

152. Vidmar, A.P., et al., *Can a Behavioral Weight-Loss Intervention Change Adolescents' Food Addiction Severity?* Childhood Obesity, 2022. **18**(3): p. 206-212.

153. Walsh, J.C., et al., *Examining the Impact of an mHealth Behavior Change Intervention With a Brief In-Person Component for Cancer Survivors With Overweight or Obesity: Randomized Controlled Trial.* JMIR mHealth and uHealth, 2021. **9**(7): p. e24915.

154. Wang, J., *A wearable sensor (Fitbit One) and text-messaging to promote physical activity and participants' level of engagement (a randomized controlled feasibility trial).* Dissertation Abstracts International: Section B: The Sciences and Engineering, 2015. **75**(12-B(E)): p. No-Specified.

155. Watt, M., et al., *A randomized control trial evaluating the impact of a web-based mind-body wellness intervention for patients with primary biliary cholangitis.* Journal of Hepatology, 2022. **77**(Supplement 1): p. S338.

156. Stiglbauer, B., S. Weber, and B. Batinic, *Does your health really benefit from using a self-tracking device? Evidence from a longitudinal randomized control trial.* Computers in Human Behavior, 2019. **94**: p. 131-139.

157. Whittemore, R., et al., *Yo puedo! A self-management group and mHealth program for low-income adults with type 2 diabetes in Mexico City.* Diabetes, 2019. **68**(Supplement 1).

158. Wiegand, B., et al., *Efficacy of a comprehensive program for reducing stress in women: a prospective, randomized trial.* Current Medical Research & Opinion, 2010. **26**(4): p. 991-1002.

159. Wilczynska, M., D.R. Lubans, and R.C. Plotnikoff, *The effects of the eCoFit RCT on depression and anxiety symptoms among adults with or at risk of Type 2 Diabetes.* Psychology, health & medicine, 2022. **27**(7): p. 1421-1430.

160. Willems, R.A., et al., *Short-term effectiveness of a web-based tailored intervention for cancer survivors on quality of life, anxiety, depression, and fatigue: randomized controlled trial.* Psycho-oncology, 2017. **26**(2): p. 222-230.

161. Williams, D.A., et al., *Internet-enhanced management of fibromyalgia: A randomized controlled trial.* Pain, 2010. **151**(3): p. 694-702.

162. Wilson, M., et al., *Engagement in online pain self-management improves pain in adults on medication-assisted behavioral treatment for opioid use disorders.* Addictive behaviors, 2018. **86**(2gw, 7603486): p. 130-137.

163. Yanez, B., et al., *Brief culturally informed smartphone interventions decrease breast cancer symptom burden among Latina breast cancer survivors.* Psycho-Oncology, 2020. **29**(1): p. 195-203.

164. Zamorano, A., et al., *Low quality of life and activity levels persist in obese endometrial cancer survivors despite participation in a behavioral weight loss intervention.* Gynecologic Oncology, 2021. **162**(Supplement 1): p. S201.

165. Zhang, Y., et al., *Effects of Online Bodyweight High-Intensity Interval Training Intervention and Health Education on the Mental Health and Cognition of Sedentary Young Females.* International journal of environmental research and public health, 2021. **18**(1).

166. Zhao, M., et al., *Effects of a Web-Based Parent-Child Physical Activity Program on Mental Health in Parents of Children with ASD.* International journal of environmental research and public health, 2021. **18**(24).

167. Zhou, H., et al., *Application of Wearables to Facilitate Virtually Supervised Intradialytic Exercise for Reducing Depression Symptoms.* Sensors (Basel, Switzerland), 2020. **20**(6).

168. Ziolkowski, S., et al., *A pilot randomized clinical trial to embed technology-enabled group-based exercise programming in the clinic: The exercise is medicine in chronic kidney disease trial.* Journal of the American Society of Nephrology, 2020. **31**((Ziolkowski, Anand, Pham) Stanford University, School of Medicine, Palo Alto, CA, United States(Bootwala, Li, Cobb, Lobelo) Emory University, School of Medicine, Atlanta, GA, United States): p. 631-632.
