# Supplementary material for: Effectiveness of Digital Lifestyle Interventions on Depression, Anxiety, Stress, and Well-Being: Systematic Review and Meta-Analysis
Source: J Med Internet Res. 2025 Mar 20;27:e56975. doi: 10.2196/56975 (PMC11969127; doi:10.2196/56975)
Supplement: Multimedia Appendix 5 [file jmir_v27i1e56975_app5.docx]

# Appendix 4.

## Figure 1. Funnel plots for meta-analyses

**
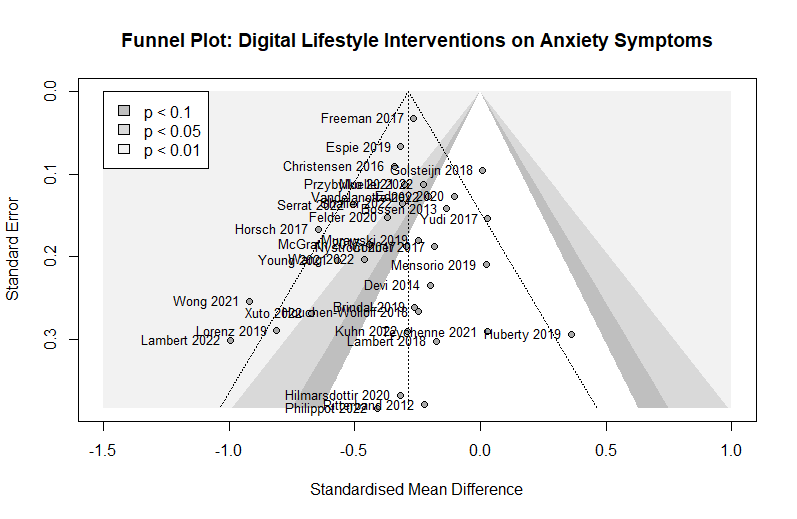

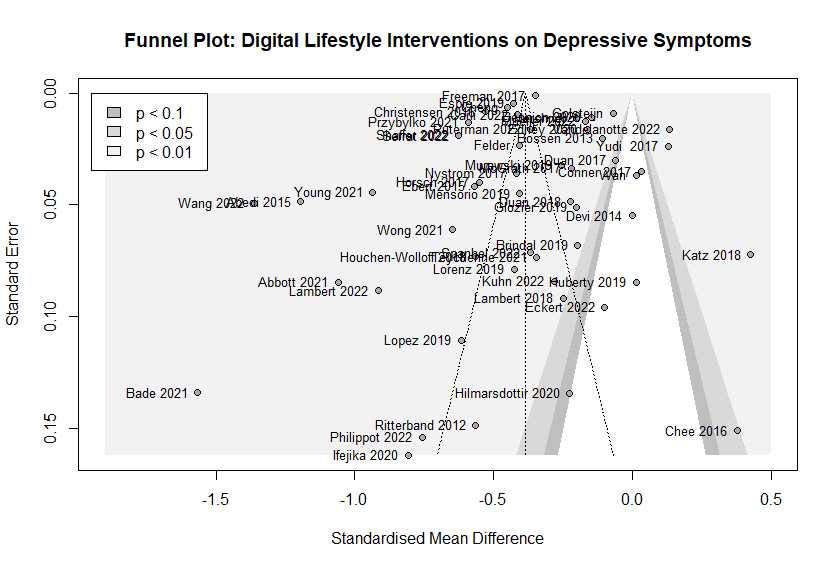

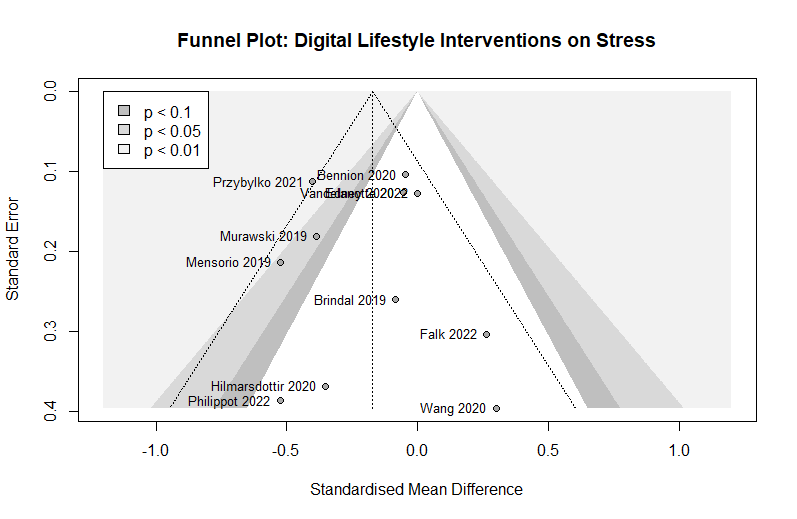

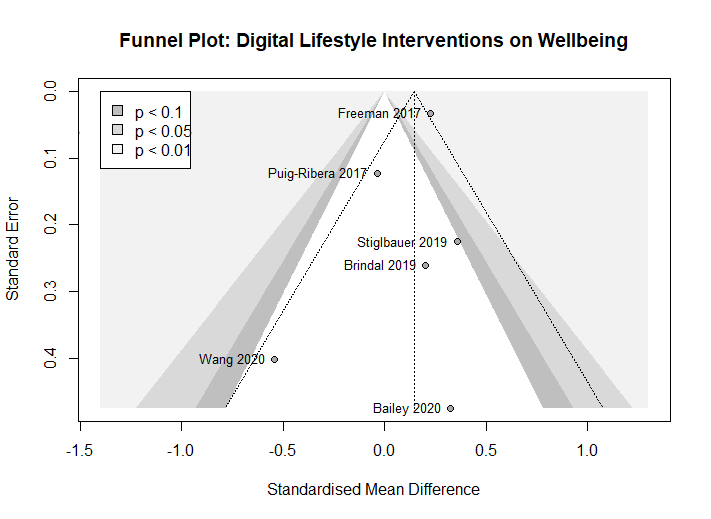
**

1. Abbott, R., et al., *Efficacy of a multimodal online lifestyle intervention for depressive symptoms and quality of life in individuals with a history of major depressive disorder.* Global Advances in Health and Medicine, 2021. **10**((Abbott) Resilient Roots: Functional and Evolutionary Medicine, Charlottesville, VA, United States(Sherwin) Midwestern University, Arizona College of Osteopathic Medicine, Glendale, AZ, United States(Klopf) Rocky Vista University, College of Osteopathic M): p. 39-40.

2. Abedi, P., P. Nikkhah, and S. Najar, *Effect of pedometer-based walking on depression, anxiety and insomnia among postmenopausal women.* Climacteric, 2015. **18**(6): p. 841-5.

3. Bade, B.C., et al., *"Randomized trial of physical activity on quality of life and lung cancer biomarkers in patients with advanced stage lung cancer: a pilot study".* BMC Cancer, 2021. **21**(1): p. 1-13.

4. Bailey, D.P., et al., *Randomised Controlled Feasibility Study of the MyHealthAvatar-Diabetes Smartphone App for Reducing Prolonged Sitting Time in Type 2 Diabetes Mellitus.* International journal of environmental research and public health, 2020. **17**(12).

5. Bennion, K.A., et al., *Impact of an Internet-Based Lifestyle Intervention on Behavioral and Psychosocial Factors During Postpartum Weight Loss.* Obesity, 2020. **28**(10): p. 1860-1867.

6. Bossen, D., et al., *Effectiveness of a web-based physical activity intervention in patients with knee and/or hip osteoarthritis: randomized controlled trial.* Journal of Medical Internet Research, 2013. **15**(11): p. e257-e257.

7. Brindal, E., et al., *A Mobile Phone App Designed to Support Weight Loss Maintenance and Well-Being (MotiMate): Randomized Controlled Trial.* Journal of Medical Internet Research, 2019. **21**(9): p. N.PAG-N.PAG.

8. Carli, V., et al., *The NEVERMIND e-health system in the treatment of depressive symptoms among patients with severe somatic conditions: A multicentre, pragmatic randomised controlled trial.* eClinicalMedicine, 2022. **48**((Carli, Petros, Hadlaczky, Vitcheva) National Centre for Suicide Research and Prevention of Mental Ill-Health, Karolinska Institutet, Stockholm, Sweden(Berchialla, Giovinazzo, Ostacoli, Settanta) Department of Clinical and Biological Sciences, Universita): p. 101423.

9. Chee, W., et al., *The effect of a culturally tailored web-based physical activity promotion program on Asian American midlife women's depressive symptoms.* Asian Pacific Island Nursing Journal, 2016. **1**(4): p. 162-173.

10. Cheng, P., et al., *Depression prevention via digital cognitive behavioral therapy for insomnia: a randomized controlled trial.* Sleep, 2019. **42**(10).

11. Christensen, H., et al., *Impact of Internet-delivered CBT for insomnia on depression symptoms in adults at-risk for major depression: Results of an RCT comparing online CBT-I versus a health website.* Sleep, 2015. **38**(SUPPL. 1): p. A236-A237.

12. Conner, T.S., et al., *Let them eat fruit! The effect of fruit and vegetable consumption on psychological well-being in young adults: A randomized controlled trial.* PloS one, 2017. **12**(2): p. e0171206.

13. Devi, R., J. Powell, and S. Singh, *A web-based program improves physical activity outcomes in a primary care angina population: randomized controlled trial.* Journal of Medical Internet Research, 2014. **16**(9): p. e186-e186.

14. Duan, Y.P., et al., *Evaluation of a Web-Based Intervention for Multiple Health Behavior Changes in Patients With Coronary Heart Disease in Home-Based Rehabilitation: Pilot Randomized Controlled Trial.* Journal of Medical Internet Research, 2018. **20**(11): p. 1-1.

15. Duan, Y.P., et al., *Web-Based Intervention for Physical Activity and Fruit and Vegetable Intake Among Chinese University Students: A Randomized Controlled Trial.* Journal of Medical Internet Research, 2017. **19**(4): p. 1-1.

16. Ebert, D.D., et al., *Restoring depleted resources: Efficacy and mechanisms of change of an internet-based unguided recovery training for better sleep and psychological detachment from work.* Health psychology : official journal of the Division of Health Psychology, American Psychological Association, 2015. **34**(Supplement): p. 1240-1251.

17. Eckert, R., et al., *A Randomized Pilot Study of Online Hatha Yoga for Physical and Psychological Symptoms Among Survivors of Allogenic Bone Marrow Transplant.* International journal of yoga therapy, 2022. **32**(2022).

18. Edney, S.M., et al., *A Social Networking and Gamified App to Increase Physical Activity: Cluster RCT.* American Journal of Preventive Medicine, 2020. **58**(2): p. e51-e62.

19. Espie, C.A., et al., *Effect of Digital Cognitive Behavioral Therapy for Insomnia on Health, Psychological Well-being, and Sleep-Related Quality of Life: A Randomized Clinical Trial.* JAMA Psychiatry, 2019. **76**(1): p. 21-30.

20. Falk, G.E., et al., *Effects of Sedentary Behavior Interventions on Mental Well-Being and Work Performance While Working from Home during the COVID-19 Pandemic: A Pilot Randomized Controlled Trial.* International journal of environmental research and public health, 2022. **19**(11).

21. Felder, J.N., et al., *Efficacy of Digital Cognitive Behavioral Therapy for the Treatment of Insomnia Symptoms Among Pregnant Women: A Randomized Clinical Trial.* JAMA Psychiatry, 2020. **77**(5): p. 484-492.

22. Freeman, D., et al., *The effects of improving sleep on mental health (OASIS): a randomised controlled trial with mediation analysis.* The lancet. Psychiatry, 2017. **4**(10): p. 749-758.

23. Glozier, N., et al., *Adjunctive Internet-delivered cognitive behavioural therapy for insomnia in men with depression: A randomised controlled trial.* Australian & New Zealand Journal of Psychiatry, 2019. **53**(4): p. 350-360.

24. Golsteijn, R.H.J., et al., *Short-term efficacy of a computer-tailored physical activity intervention for prostate and colorectal cancer patients and survivors: a randomized controlled trial.* The international journal of behavioral nutrition and physical activity, 2018. **15**(1): p. 106.

25. Hershner, S. and L.M. O'Brien, *The impact of a randomized sleep education intervention for college students.* Journal of Clinical Sleep Medicine, 2018. **14**(3): p. 337-347.

26. Hilmarsdottir, E., A.K. Sigurdardottir, and R.H. Arnardottir, *A Digital Lifestyle Program in Outpatient Treatment of Type 2 Diabetes: A Randomized Controlled Study.* Journal of Diabetes Science and Technology, 2020. **15**(5): p. 1134-1141.

27. Horsch, C.H.G., et al., *Mobile Phone-Delivered Cognitive Behavioral Therapy for Insomnia: A Randomized Waitlist Controlled Trial.* Journal of Medical Internet Research, 2017. **19**(4): p. 1-1.

28. Houchen-Wolloff, L., et al., *Web-based cardiac RE habilitatio N alternative for those declining or dropping out of conventional rehabilitation: Results of the WREN feasibility randomised controlled trial.* Open Heart, 2018. **5**(2): p. e000860.

29. Huberty, J., et al., *Online yoga in myeloproliferative neoplasm patients: results of a randomized pilot trial to inform future research.* BMC Complementary & Alternative Medicine, 2019. **19**(1): p. N.PAG-N.PAG.

30. Ifejika, N.L., et al., *Use of a Smartphone-Based Mobile App for Weight Management in Obese Minority Stroke Survivors: Pilot Randomized Controlled Trial With Open Blinded End Point.* JMIR mHealth and uHealth, 2020. **8**(4): p. e17816.

31. Joutsenniemi, K., et al., *E-mail-based exercises in happiness, physical activity and readings: A randomized trial on 3274 Finns.* African Journal of Psychiatry, 2014. **17**(5): p. 1-13.

32. Katz, P., et al., *Physical Activity to Reduce Fatigue in Rheumatoid Arthritis: A Randomized Controlled Trial.* Arthritis Care & Research, 2018. **70**(1): p. 1-10.

33. Kuhn, E., et al., *A Pilot Randomized Controlled Trial of the Insomnia Coach Mobile App to Assess Its Feasibility, Acceptability, and Potential Efficacy.* Behavior Therapy, 2022. **53**(3): p. 440-457.

34. Lambert, S.D., et al., *Feasibility, Acceptability, and Clinical Significance of a Dyadic, Web-Based, Psychosocial and Physical Activity Self-Management Program (TEMPO) Tailored to the Needs of Men with Prostate Cancer and Their Caregivers: A Multi-Center Randomized Pilot Trial.* Current Oncology, 2022. **29**(2): p. 785-804.

35. Lambert, J.D., et al., *Web-Based Intervention Using Behavioral Activation and Physical Activity for Adults With Depression (The eMotion Study): Pilot Randomized Controlled Trial.* Journal of medical Internet research, 2018. **20**(7): p. e10112.

36. Lopez, R., et al., *French Language Online Cognitive Behavioral Therapy for Insomnia Disorder: A Randomized Controlled Trial.* Frontiers in Neurology, 2019. **10**((Lopez, Evangelista, Barateau, Chenini, Bosco, Dauvilliers) Centre National de Reference Narcolepsie Hypersomnies, Unite des Troubles du Sommeil, Departement de Neurologie, Hopital Gui-de-Chauliac, Montpellier, France(Lopez, Evangelista, Barateau, Beziat): p. 1273.

37. Lorenz, N., et al., *Randomized Controlled Trial to Test the Efficacy of an Unguided Online Intervention with Automated Feedback for the Treatment of Insomnia.* Behavioural and cognitive psychotherapy, 2019. **47**(3): p. 287-302.

38. McGrath, E.R., et al., *Sleep to Lower Elevated Blood Pressure: A Randomized Controlled Trial (SLEPT).* American journal of hypertension, 2017. **30**(3): p. 319-327.

39. Mensorio, M.S., et al., *Analysis of the efficacy of an internet-based self-administered intervention ("Living Better") to promote healthy habits in a population with obesity and hypertension: An exploratory randomized controlled trial.* International Journal of Medical Informatics, 2019. **124**: p. 13-23.

40. Mueller, J., et al., *Supporting Weight Management during COVID-19: A Randomized Controlled Trial of a Web-Based, ACT-Based, Guided Self-Help Intervention.* Obesity facts, 2022. **15**(4): p. 550-559.

41. Murawski, B., et al., *Efficacy of an m-Health Physical Activity and Sleep Health Intervention for Adults: A Randomized Waitlist-Controlled Trial.* American Journal of Preventive Medicine, 2019. **57**(4): p. 503-514.

42. Nystrom, M.B.T., et al., *Behavioral activation versus physical activity via the internet: A randomized controlled trial.* Journal of affective disorders, 2017. **215**(h3v, 7906073): p. 85-93.

43. Philippot, A., et al., *Feasibility of Online High-Intensity Interval Training (HIIT) on Psychological Symptoms in Students in Lockdown During the COVID-19 Pandemic: A Randomized Controlled Trial.* Frontiers in Psychiatry, 2022. **13**((Philippot, de Timary, De Volder, Bleyenheuft) Institute of Neuroscience, Universite catholique de Louvain, Brussels, Belgium(Philippot, Dubois) Psychiatric Hospital Area+/Epsylon ASBL, Brussels, Belgium(Moulin, Charon, Balestra, Lambrechts) Environmental): p. 904283.

44. Przybylko, G., et al., *The effectiveness of an online interdisciplinary intervention for mental health promotion: a randomized controlled trial.* BMC psychology, 2021. **9**(1): p. 77.

45. Puig-Ribera, A., et al., *Impact of a workplace 'sit less, move more' program on efficiency-related outcomes of office employees.* BMC public health, 2017. **17**(1): p. 455.

46. Puterman, E., et al., *COVID-19 Pandemic and Exercise (COPE) trial: a multigroup pragmatic randomised controlled trial examining effects of app-based at-home exercise programs on depressive symptoms.* British journal of sports medicine, 2022. **56**(10): p. 546-552.

47. Ritterband, L.M., et al., *Initial evaluation of an Internet intervention to improve the sleep of cancer survivors with insomnia.* Psycho-Oncology, 2012. **21**(7): p. 695-705.

48. Savard, J., et al., *Is a video-based cognitive behavioral therapy for insomnia as efficacious as a professionally administered treatment in breast cancer? Results of a randomized controlled trial.* Sleep, 2014. **37**(8): p. 1305-14.

49. Serrat, M., et al., *Effectiveness of two video-based multicomponent treatments for fibromyalgia: The added value of cognitive restructuring and mindfulness in a three-arm randomised controlled trial.* Behaviour research and therapy, 2022. **158**(9kp, 0372477): p. 104188.

50. Shaffer, K.M., et al., *Effects of an internet-delivered insomnia intervention for older adults: A secondary analysis on symptoms of depression and anxiety.* Journal of Behavioral Medicine, 2022. **45**(5): p. 728-738.

51. Spanhel, K., et al., *Engaging Refugees With a Culturally Adapted Digital Intervention to Improve Sleep: A Randomized Controlled Pilot Trial.* Frontiers in Psychiatry, 2022. **13**((Spanhel, Hovestadt, Bengel, Sander) Department of Rehabilitation Psychology and Psychotherapy, Institute of Psychology, University of Freiburg, Freiburg, Germany(Lehr) Department of Health Psychology, Institute of Psychology, Leuphana University Luenebur): p. 832196.

52. Stiglbauer, B., S. Weber, and B. Batinic, *Does your health really benefit from using a self-tracking device? Evidence from a longitudinal randomized control trial.* Computers in Human Behavior, 2019. **94**: p. 131-139.

53. Teychenne, M., et al., *Mums on the Move: A pilot randomised controlled trial of a home-based physical activity intervention for mothers at risk of postnatal depression.* Midwifery, 2021. **93**: p. N.PAG-N.PAG.

54. Vandelanotte, C., et al., *Impact of a web-based personally tailored physical activity intervention on depression, anxiety, stress and quality of life: Secondary outcomes from a randomized controlled trial.* Mental Health and Physical Activity, 2022. **23**((Vandelanotte, Rebar, Alley, Schoeppe, To) Central Queensland University, Appleton Institute, Physical Activity Research Group, Rockhampton, QLD 4702, Australia(Duncan) The University of Newcastle, College of Health, Medicine, and Wellbeing, School of Med): p. 100477.

55. Wan, E.S., et al., *Promoting physical activity in COPD: Insights from a randomized trial of a web-based intervention and pedometer use.* Respiratory medicine, 2017. **130**(8908438, rme): p. 102-110.

56. Wang, L., et al., *Transtheoretical model-based mobile health application for PCOS.* Reproductive Health, 2022. **19**(1): p. 1-10.

57. Wang, F. and S. Boros, *Effects of a pedometer-based walking intervention on young adults' sleep quality, stress and life satisfaction: Randomized controlled trial.* Journal of Bodywork & Movement Therapies, 2020. **24**(4): p. 286-292.

58. Wong, V.W.-H., et al., *Smartphone-delivered multicomponent lifestyle medicine intervention for depressive symptoms: A randomized controlled trial.* Journal of consulting and clinical psychology, 2021. **89**(12): p. 970-984.

59. Young, M.D., et al., *Impact of a self-guided, eHealth program targeting weight loss and depression in men: A randomized trial.* Journal of consulting and clinical psychology, 2021. **89**(8): p. 682-694.

60. Xuto, P., et al., *Effect of Receiving Text Messages on Health Care Behavior and State Anxiety of Thai Pregnant Women: A Randomized Controlled Trial.* Int J Community Based Nurs Midwifery, 2022. **10**(1): p. 18-29.

61. Yudi, M., et al., *Smartphone-based, early cardiac rehabilitation in patients with acute coronary syndromes [smart-rehab trial]: A randomised controlled trial.* Heart Lung and Circulation, 2017. **26**(Supplement 2): p. S349.
