# Supplementary material for: Effectiveness of Digital Lifestyle Interventions on Depression, Anxiety, Stress, and Well-Being: Systematic Review and Meta-Analysis
Source: J Med Internet Res. 2025 Mar 20;27:e56975. doi: 10.2196/56975 (PMC11969127; doi:10.2196/56975)
Supplement: Multimedia Appendix 6 [file jmir_v27i1e56975_app6.docx]

# Appendix 5.

## Table 3. Meta-regression analyses for all outcomes

| **Moderator** | **k** | **β** | **95% CI** | | **P value** | **R^2^ (%)** |
| --- | --- | --- | --- | --- | --- | --- |
| ***Depression*** | | | | | | |
| PEDro score | 53 | 0.01 | -0.064 | 0.075 | 0.87 | 0 |
| Duration of intervention | 53 | -0.01 | -0.015 | 0.005 | 0.30 | 0 |
| Publication year | 53 | -0.04 | -0.075 | 0.003 | 0.07 | 5.10 |
| ***Anxiety*** | | | | | | |
| PEDro score | 35 | -0.01 | -0.07 | 0.04 | 0.57 | 0 |
| Duration of intervention | 35 | -0.002 | -0.01 | 0.01 | 0.67 | 0 |
| Publication year | 35 | -0.02 | -0.05 | 0.01 | 0.13 | 0 |
| ***Stress*** | | | | | | |
| PEDro score | 11 | -0.05 | -0.37 | 0.26 | 0.71 | 0 |
| Duration of intervention | 11 | 0.003 | -0.007 | 0.01 | 0.48 | 0 |
| Publication year | 11 | 0.07 | -0.09 | 0.22 | 0.35 | 0 |
| ***Wellbeing*** | | | | | | |
| PEDro score | 6 | 0.04 | -0.08 | 0.15 | 0.40 | 0.35 |
| Duration of intervention | 6 | -0.01 | -0.05 | 0.03 | 0.45 | 58.2 |
| Publication year | 6 | -0.01 | -0.27 | 0.25 | 0.91 | 0 |
