# Supplementary material for: Effectiveness of Digital Lifestyle Interventions on Depression, Anxiety, Stress, and Well-Being: Systematic Review and Meta-Analysis
Source: J Med Internet Res. 2025 Mar 20;27:e56975. doi: 10.2196/56975 (PMC11969127; doi:10.2196/56975)
Supplement: Multimedia Appendix 7 [file jmir_v27i1e56975_app7.docx]

# Appendix 6.

## Table 4. Sensitivity analyses for all meta-analyses

|  | | **k** | **SMD** | **95% CI** | **P value** |
| --- | --- | --- | --- | --- | --- |
| ***Depression*** | | | | | |
| All studies | | 53 | -0.37 | -0.46, -0.27 | <.001 |
| Removing outliers | | 44 | -0.34 | -0.41, -0.28 | <.001 |
| Removing fair/poor quality studies | | 48 | -0.38 | -0.49, -0.28 | <.001 |
| Removing small studies (N=<100) | | 25 | -0.28 | -0.38, -0.19 | <.001 |
| Removing studies with >25% attrition | | 35 | -0.4 | -0.54, -0.26 | <.001 |
| ***Anxiety*** | | | | | |
| All studies | | 35 | -0.29 | -0.36, -0.21 | <.001 |
| Removing outliers | | 32 | -0.28 | -0.33, -0.23 | <.001 |
| Removing fair/poor quality studies | | 34 | -0.29 | -0.37, -0.21 | <.001 |
| Removing small studies (N=<100) | | 18 | -0.26 | -0.34, -0.19 | <.001 |
| Removing studies with >25% attrition | | 23 | -0.31 | -0.43, -0.18 | <.001 |
| ***Stress*** |  | | |  | |
| All studies | | 11 | -0.17 | -0.33, -0.0 | <.001 |
| Removing outliers | | - |  |  |  |
| Removing fair/poor quality studies | | 10 | -0.19 | -0.35, -0.02 | 0.03 |
| Removing small studies (N=<100) | | 5 | -0.16 | -0.41, 0.08 | 0.13 |
| Removing studies with >25% attrition | | 6 | -0.11 | -0.3, 0.09 | 0.22 |
| ***Wellbeing*** |  | | |  | |
| All studies | | 6 | 0.14 | -0.08, 0.37 | 0.15 |
| Removing outliers | | - |  |  |  |
| Removing fair/poor quality studies | | 4 | 0.15 | -0.07, 0.37 | 0.12 |
| Removing small studies (N=<100) | | 2 | 0.12 | -1.51, 1.75 | 0.52 |
| Removing studies with >25% attrition | | 2 | 0.35 | 0.19, 0.51 | 0.95 |
